# On the attenuation and amplification of molecular noise in genetic regulatory networks

**DOI:** 10.1186/1471-2105-7-52

**Published:** 2006-02-02

**Authors:** Bor-Sen Chen, Yu-Chao Wang

**Affiliations:** 1 Lab of Control and Systems Biology, Department of Electrical Engineering, National Tsing Hua University, Hsinchu, 300, Taiwan

## Abstract

**Background:**

Noise has many important roles in cellular genetic regulatory functions at the nanomolar scale. At present, no good theory exists for identifying all possible mechanisms of genetic regulatory networks to attenuate the molecular noise to achieve regulatory ability or to amplify the molecular noise to randomize outcomes to the advantage of diversity. Therefore, the noise filtering of genetic regulatory network is an important topic for gene networks under intrinsic fluctuation and extrinsic noise.

**Results:**

Based on stochastic dynamic regulation equation, the intrinsic fluctuation in reaction rates is modeled as a state-dependent stochastic process, which will influence the stability of gene regulatory network, especially, with low concentrations of reacting species. Then the mechanisms of genetic regulatory network to attenuate or amplify extrinsic fluctuation are revealed from the nonlinear stochastic filtering point of view. Furthermore, a simple measure of attenuation level or amplification level of extrinsic noise for genetic regulatory networks is also introduced by nonlinear robust filtering method. Based on the global linearization scheme, a convenient method is introduced to measure noise attenuation or amplification for each gene of the nonlinear stochastic regulatory network by solving a set of filtering problems, which correspond to a set of linearized stochastic regulatory networks. Finally, by the proposed methods, several simulation examples of genetic regulatory networks are given to measure their robust stability under intrinsic fluctuations, and to estimate the genes' attenuation and amplification levels under extrinsic noises.

**Conclusion:**

In this study, a stochastic nonlinear dynamic model is developed for genetic regulatory networks under intrinsic fluctuation and extrinsic noise. By the method we proposed, we could determine the robust stability under intrinsic fluctuations and identify the genes that are significantly affected by extrinsic noises, which we call the weak structure of the network. This method will be potential for robust gene circuit design in future, on which a drug design could be based.

## Background

Molecular noise has been shown to play many roles in the biological functions of genetic regulatory networks, including noise-driven divergence of cell fates and population heterogeneity, noise-induced amplification of signals, generation of errors in DNA replication leading to mutation and evolution, and maintenance of the quantitative individual of cells [1]. Other cellular processes influenced by noise include ion-channel gating [2], neural firing [3], developmental module [4,5], cytoskeleton dynamics [6] and motors [7]. Phase variation in pathogenic bacteria, where cells alternate randomly between expressing certain genes and silencing others, is thought to be a form of cultivated noise [8]. These molecular-level noisy phenomena are deeply rooted in the statistical mechanical behavior of so-called nanoscale chemical systems, where concentrations of reacting species are extremely low and, consequently, fluctuations (noises) in the reaction rates are large [9]. Even though the molecular fluctuations leading to phase variation seem random in the individual, regulatory factors tune the variation to ensure mean levels of heterogeneity for the population, i.e., the random noises can be shown to be filtered or attenuated by the genetic regulatory networks [1].

Since molecular events in cells are subject to significant thermal fluctuations and noisy process with transcriptional control, alternative splicing, translation, diffusion and chemical modification reaction, thus gene expression is best viewed as a stochastic process. Many observations suggest that molecular events underlying cellular physiology are subject to fluctuations and have lead to the proposal of a stochastic model for gene expressions and biofunctions [9-13].

Noise attenuation can be considered from the signal processing perspective [1,14,15]. From this perspective, a pathway is viewed as an analog filter in terms of its frequency response. In terms of signal processing, these pathways function as low-pass filters to transduce low-frequency signal and to attenuate high-frequency noise [14,16]. Negative feedback schemes are the most common noise-attenuation regulatory mechanism, which make a gene network robust to gene expression noise [17-19]. Intrinsic chemical damping, integral feedback and redundancy have also found to be efficient noise filtering schemes in genetic regulatory systems [9,20].

Some cellular processes amplify or exploit noise in some sense, rather than just controlling or eliminating it. These processes fall into two mechanisms – one gives rise to population heterogeneity (and thus produces diversity with fitness advantage) [19], and the other uses noise to attenuate noise. Several results of isogenic heterogeneity illustrate how intrinsic molecular noise is used to generate diversity and how intrinsic molecular noise results in phenotypic variation and cellular differentiation [19,21]. How gene expression noise can lead to interesting dynamics in gene regulatory networks in found in [22]. The *fim *network regulates phase variation of type 1 pili uropathic *E. coli *. This system provides an example of how integrated regulatory modules in a network can function to both shape and filter noise, thereby creating environmentally tuned heterogeneity in a cell population [8,23]. Positive feedback can amplify the effect of noise and population heterogeneity by autocatalytic mechanisms [19,24-26]. In addition to generating heterogeneous populations, cells also use noise to filter noise. Although in most systems, noise degrades a signal, when certain nonlinear effects are present, noise actually enhance a signal; for example, stochastic resonance [27].

Some elementary mechanisms for noise attenuation and amplification are tractable because they appear simple and identifiable. However, elementary mechanisms typically do not function in isolation, but rather interact in complex networks involving multiple feedback loops. Although it is straightforward to understand how a simple feedback loop shapes noise, it is far more difficult to understand the composite noise shaping behavior of multiple mechanisms interconnected in complex regulatory networks [28]. Recently, several computational models have been proposed for genetic regulatory networks that control circadian rhythms with regular oscillations in the presence of noise, using both deterministic and stochastic model [29-31]. The stochastic model is able to produce regular oscillations, whereas the deterministic model can not [31], suggesting that the regulatory networks may utilize molecular fluctuations to their advantage.

Several examples have shown that noise attenuation arises from systematic properties of networks rather than a single mechanism. What specific mechanisms confer robust functionality in the presence of noise? It is clear that large, complex networks are able to function reliably despite inherent noise attributable to molecular fluctuations. Apparently, noise attenuation arises from complex network mechanisms involving multiple feedback loops [1,32].

Although theoretical and computational tools exist for analyzing the properties of a given genetic regulatory network, no good theory exists for identifying noise attenuation and amplification mechanisms of the networks. Although noise attenuation and amplification examples abound, how cells are able to manipulate biochemical noise remain unknown. By what means do regulatory networks attenuate the noise? And how and why do networks amplify noise? As pointed out by Rao *et al. *[1], these questions present one of the most challenging and fascinating problems for systems biologists, since they open questions in physiology, development and evolutionary biology. The answer likely resides in complex gene regulatory networks. Stochastic dynamic models are the ideal tools for such investigations, because they allow us to describe quantitatively the current states of network structure and component interactions to explore the network stochastic dynamics under intrinsic fluctuations and extrinsic noises.

Recently, a robustness measure for biochemical networks has been discussed at steady states by the singular value of the system matrix for a S-system model with deterministic parameter perturbations [33]. However, using this approach, it is not easy to discuss the robustness of nonlinear gene regulatory networks under stochastic intrinsic and extrinsic noises. In this study, based on a stochastic dynamic model of genetic regulatory networks with intrinsic and extrinsic noises, the robust stability to tolerate the intrinsic noise is initially revealed from the Lyapunov (energy) function point of view, and then a measure of the extrinsic noise attenuation level or amplification level of regulatory networks is developed from the H_∞ _filtering point of view. In the last two decades, H_∞ _control theory has been developed to efficiently attenuate the effect of extrinsic disturbance from the L_2_-gain point of view [34-36]. Recently, noise attenuation of nonlinear stochastic systems has been developed for state estimation from the H_∞ _filtering perspective [15]. These attenuation methods for extrinsic disturbances could be employed with some modifications as theoretical and computational bases for noise attenuation and amplification of gene regulatory networks. For the simplicity of illustration, a linear stochastic model with extrinsic and intrinsic noises is given at first to discuss the noise shaping of genetic regulatory network. Then the nonlinear stochastic equation is introduced to describe a general genetic regulatory network under extrinsic and intrinsic noises.

In this study, the intrinsic noises due to parametric fluctuations are modeled as state dependent noise, which will influence the stability of the gene regulatory network. The robust stability to tolerate these intrinsic parameter fluctuations by gene regulatory networks is discussed by the Lyapunov stability theory of nonlinear stochastic systems. We need to solve a Hamilton-Jacobi inequality (HJI) to measure the robustness of stability of nonlinear gene regulatory networks [37]. The ability to attenuate the extrinsic noises of nonlinear gene regulatory networks is measured based on the nonlinear H_∞ _filtering theory [15]. In order to avoid solving HJI in nonlinear stochastic gene regulatory networks, based on the global linearization technique, a set of linear matrix inequalities (LMIs) [38], which can be efficiently solved by LMI Toolbox of Matlab, are employed to replace HJI. This allows us to measure the robust stability with respect to parametric fluctuations and to estimate the attenuation level or amplification level of nonlinear stochastic gene regulatory networks under intrinsic extrinsic noises.

Finally, a genetic regulatory network under intrinsic and extrinsic noises is considered for the illustration and the performance confirmation of the proposed method. According to intense simulation results, the proposed attenuation and amplification schemes could be a satisfactory method to interpret the stability robustness and noise filtering of each gene in a gene regulatory network under intrinsic fluctuations and extrinsic noises from the systems biology perspective.

## Results

### Stochastic system model and noise filtering

#### Network robust stability under intrinsic fluctuation

**Figure 1:**
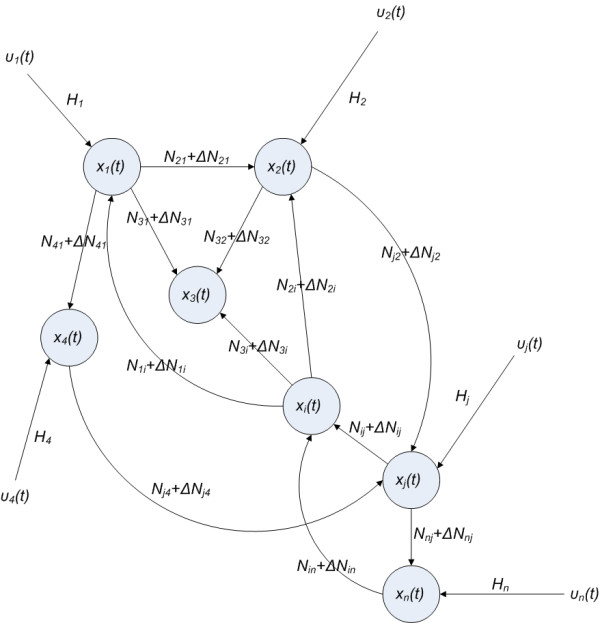
The linear genetic regulatory network with intrinsic fluctuation Δ*N*_*ij *_and extrinsic fluctuation *υ*_*i*_(*t*).

Initially, for the convenience of illustration, we consider only the following linear biochemical dynamics of a n-genes genetic regulatory network in Figure 1

dx(t)dt=Nx(t)     (1)
 MathType@MTEF@5@5@+=feaafiart1ev1aaatCvAUfKttLearuWrP9MDH5MBPbIqV92AaeXatLxBI9gBaebbnrfifHhDYfgasaacH8akY=wiFfYdH8Gipec8Eeeu0xXdbba9frFj0=OqFfea0dXdd9vqai=hGuQ8kuc9pgc9s8qqaq=dirpe0xb9q8qiLsFr0=vr0=vr0dc8meaabaqaciaacaGaaeqabaqabeGadaaakeaadaWcaaqaaiabdsgaKjabdIha4jabcIcaOiabdsha0jabcMcaPaqaaiabdsgaKjabdsha0baacqGH9aqpcqWGobGtcqWG4baEcqGGOaakcqWG0baDcqGGPaqkcaWLjaGaaCzcamaabmaabaGaeGymaedacaGLOaGaayzkaaaaaa@3FEF@

where the concentration vector *x*(*t*) and stoichiometric matrix *N *are given by

x(t)=[x1(t)x2(t)⋮xn(t)],N=[N11…N1n⋮Nij⋮Nn1…Nnn]
 MathType@MTEF@5@5@+=feaafiart1ev1aaatCvAUfKttLearuWrP9MDH5MBPbIqV92AaeXatLxBI9gBaebbnrfifHhDYfgasaacH8akY=wiFfYdH8Gipec8Eeeu0xXdbba9frFj0=OqFfea0dXdd9vqai=hGuQ8kuc9pgc9s8qqaq=dirpe0xb9q8qiLsFr0=vr0=vr0dc8meaabaqaciaacaGaaeqabaqabeGadaaakeaacqWG4baEcqGGOaakcqWG0baDcqGGPaqkcqGH9aqpfaqabeqacaaabaWaamWaaeaafaqabeabbaaaaeaacqWG4baEdaWgaaWcbaGaeGymaedabeaakiabcIcaOiabdsha0jabcMcaPaqaaiabdIha4naaBaaaleaacqaIYaGmaeqaaOGaeiikaGIaemiDaqNaeiykaKcabaGaeSO7I0eabaGaemiEaG3aaSbaaSqaaiabd6gaUbqabaGccqGGOaakcqWG0baDcqGGPaqkaaaacaGLBbGaayzxaaGaeiilaWcabaGaemOta4Kaeyypa0ZaamWaaeaafaqabeWadaaabaGaemOta40aaSbaaSqaaiabigdaXiabigdaXaqabaaakeaacqWIVlctaeaacqWGobGtdaWgaaWcbaGaeGymaeJaemOBa4gabeaaaOqaaiabl6Uinbqaaiabd6eaonaaBaaaleaacqWGPbqAcqWGQbGAaeqaaaGcbaGaeSO7I0eabaGaemOta40aaSbaaSqaaiabd6gaUjabigdaXaqabaaakeaacqWIVlctaeaacqWGobGtdaWgaaWcbaGaemOBa4MaemOBa4gabeaaaaaakiaawUfacaGLDbaaaaaaaa@67A8@

in which *x*_*i*_(*t*) denotes the concentration of the *i*th gene, and *N*_*ij *_denotes the interaction between gene *j *and gene *i*.

Suppose the linear genetic regulatory network suffers intrinsic molecular fluctuations mainly due to stochastic thermal fluctuation so that stoichiometric matrix *N *is perturbed as *N *+ Δ*N*, where the random components of the perturbation Δ*N *could be modeled by a stochastic Wiener process *ω*(*t*) on a probability space (Ω, *p*), which is a mathematical description of the so-called Brownian motion [39].

In intrinsic fluctuations, the perturbed dynamics of the nominal genetic regulatory network in equation (1) could be modeled as the following linear stochastic equation

*dx*(*t*) = *Nx*(*t*)*dt *+ *Mx*(*t*)*dω*(*t*)     (2)

This is a standard linear stochastic dynamic equation with state dependent noise. *Mx*(*t*)*dω*(*t*) denotes the term due to the intrinsic fluctuation Δ*Nx*(*t*), where the stochastic property of fluctuation Δ*N *is extracted out to the standard Wiener process (or Brownian motion) *ω*(*t*) (which, roughly speaking, is integral of white noise) using *E*{|*ω*(*t*) - *ω*(*τ*)|^2 ^}= *σ*^2 ^|*t* - *τ*| with unit covariance *σ*^2 ^= 1 For example, ΔN=[ΔN11(t)…ΔN1n(t)⋮ΔNij(t)⋮ΔNn1(t)…ΔNnn(t)]=[M11…M1n⋮Mij⋮Mn1…Mnn]n(t)=Mn(t)
 MathType@MTEF@5@5@+=feaafiart1ev1aaatCvAUfKttLearuWrP9MDH5MBPbIqV92AaeXatLxBI9gBaebbnrfifHhDYfgasaacH8akY=wiFfYdH8Gipec8Eeeu0xXdbba9frFj0=OqFfea0dXdd9vqai=hGuQ8kuc9pgc9s8qqaq=dirpe0xb9q8qiLsFr0=vr0=vr0dc8meaabaqaciaacaGaaeqabaqabeGadaaakeaacqGHuoarcqWGobGtcqGH9aqpdaWadaqaauaabeqadmaaaeaacqGHuoarcqWGobGtdaWgaaWcbaGaeGymaeJaeGymaedabeaakiabcIcaOiabdsha0jabcMcaPaqaaiabl+Uimbqaaiabgs5aejabd6eaonaaBaaaleaacqaIXaqmcqWGUbGBaeqaaOGaeiikaGIaemiDaqNaeiykaKcabaGaeSO7I0eabaGaeyiLdqKaemOta40aaSbaaSqaaiabdMgaPjabdQgaQbqabaGccqGGOaakcqWG0baDcqGGPaqkaeaacqWIUlstaeaacqGHuoarcqWGobGtdaWgaaWcbaGaemOBa4MaeGymaedabeaakiabcIcaOiabdsha0jabcMcaPaqaaiabl+Uimbqaaiabgs5aejabd6eaonaaBaaaleaacqWGUbGBcqWGUbGBaeqaaOGaeiikaGIaemiDaqNaeiykaKcaaaGaay5waiaaw2faaiabg2da9maadmaabaqbaeqabmWaaaqaaiabd2eannaaBaaaleaacqaIXaqmcqaIXaqmaeqaaaGcbaGaeS47IWeabaGaemyta00aaSbaaSqaaiabigdaXiabd6gaUbqabaaakeaacqWIUlstaeaacqWGnbqtdaWgaaWcbaGaemyAaKMaemOAaOgabeaaaOqaaiabl6Uinbqaaiabd2eannaaBaaaleaacqWGUbGBcqaIXaqmaeqaaaGcbaGaeS47IWeabaGaemyta00aaSbaaSqaaiabd6gaUjabd6gaUbqabaaaaaGccaGLBbGaayzxaaGaemOBa4MaeiikaGIaemiDaqNaeiykaKIaeyypa0Jaemyta0KaemOBa4MaeiikaGIaemiDaqNaeiykaKcaaa@8C4D@ , where *M*_*ij *_denotes the deterministic part (amplitude) of fluctuation and *n*(*t*) is a white Gaussian noise with zero mean and unit variance to denote the stochastic part of fluctuation, i.e., the stochastic part of fluctuation is absorbed to *n*(*t*) with *dω*(*t*) = *n*(*t*)*dt *[15,36,37]. If some components *ij *of *N *are free of intrinsic fluctuation, then the corresponding *M*_*ij *_should be equal to zero. And the covariance of Δ*N*_*ij*_(*t*) is Cov(*M*_*ij*_*n*(*t*), *M*_*ij*_*n(τ)) *= Mij2
 MathType@MTEF@5@5@+=feaafiart1ev1aaatCvAUfKttLearuWrP9MDH5MBPbIqV92AaeXatLxBI9gBaebbnrfifHhDYfgasaacH8akY=wiFfYdH8Gipec8Eeeu0xXdbba9frFj0=OqFfea0dXdd9vqai=hGuQ8kuc9pgc9s8qqaq=dirpe0xb9q8qiLsFr0=vr0=vr0dc8meaabaqaciaacaGaaeqabaqabeGadaaakeaacqWGnbqtdaqhaaWcbaGaemyAaKMaemOAaOgabaGaeGOmaidaaaaa@31A6@*δ *(*t *- *τ*), where *δ *(*t*) is the impulse function.

In the conventional notation of engineering and system science, the stochastic dynamic equation could be represented by [39]

ddtx(t)=Nx(t)+Mx(t)n(t)     (3)
 MathType@MTEF@5@5@+=feaafiart1ev1aaatCvAUfKttLearuWrP9MDH5MBPbIqV92AaeXatLxBI9gBaebbnrfifHhDYfgasaacH8akY=wiFfYdH8Gipec8Eeeu0xXdbba9frFj0=OqFfea0dXdd9vqai=hGuQ8kuc9pgc9s8qqaq=dirpe0xb9q8qiLsFr0=vr0=vr0dc8meaabaqaciaacaGaaeqabaqabeGadaaakeaadaWcaaqaaiabdsgaKbqaaiabdsgaKjabdsha0baacqWG4baEcqGGOaakcqWG0baDcqGGPaqkcqGH9aqpcqWGobGtcqWG4baEcqGGOaakcqWG0baDcqGGPaqkcqGHRaWkcqWGnbqtcqWG4baEcqGGOaakcqWG0baDcqGGPaqkcqWGUbGBcqGGOaakcqWG0baDcqGGPaqkcaWLjaGaaCzcamaabmaabaGaeG4mamdacaGLOaGaayzkaaaaaa@4B1C@

where *n*(*t*) ≡ *dω*(*t*)/*dt *denotes a normalized white Gaussian noise with unit covariance. Since *ω*(*t*) is a stochastic process, *x*(*t*) in equation (2) or (3) is also a stochastic process. Actually, the stochastic dynamic equations of genetic regulatory networks are always nonlinear. In order to meet the nonlinear stochastic regulatory networks, equation (3) should be generalized as the following Langevin equation [1]

*dx*(*t*) = *N*(*x*)*dt *+ *M*(*x*)*dω*(*t*)     (4)

where *N(x) *denotes the nonlinear interaction equation of nonlinear genetic regulatory network and *M*(*x*)*dω*(*t*) is due to nonlinear intrinsic fluctuation.

Remark: (i) At different operation points, the linearization of nonlinear stochastic regulatory network of equation (4) will be of the form in equation (3), i.e., at an operation point *x *= *x*_0_, N=∂N(x)∂x|x=x0
 MathType@MTEF@5@5@+=feaafiart1ev1aaatCvAUfKttLearuWrP9MDH5MBPbIqV92AaeXatLxBI9gBaebbnrfifHhDYfgasaacH8akY=wiFfYdH8Gipec8Eeeu0xXdbba9frFj0=OqFfea0dXdd9vqai=hGuQ8kuc9pgc9s8qqaq=dirpe0xb9q8qiLsFr0=vr0=vr0dc8meaabaqaciaacaGaaeqabaqabeGadaaakeaacqWGobGtcqGH9aqpdaabcaqaamaalaaabaacciGae8NaIyRaemOta4KaeiikaGIaemiEaGNaeiykaKcabaGae8NaIyRaemiEaGhaaaGaayjcSdqbaeqabiqaaaqaaaqaaiabdIha4jabg2da9iabdIha4naaBaaaleaaieGacqGFWaamaeqaaaaaaaa@3E30@ and M=∂M(x)∂x|x=x0
 MathType@MTEF@5@5@+=feaafiart1ev1aaatCvAUfKttLearuWrP9MDH5MBPbIqV92AaeXatLxBI9gBaebbnrfifHhDYfgasaacH8akY=wiFfYdH8Gipec8Eeeu0xXdbba9frFj0=OqFfea0dXdd9vqai=hGuQ8kuc9pgc9s8qqaq=dirpe0xb9q8qiLsFr0=vr0=vr0dc8meaabaqaciaacaGaaeqabaqabeGadaaakeaacqWGnbqtcqGH9aqpdaabcaqaamaalaaabaacciGae8NaIyRaemyta0KaeiikaGIaemiEaGNaeiykaKcabaGae8NaIyRaemiEaGhaaaGaayjcSdqbaeqabiqaaaqaaaqaaiabdIha4jabg2da9iabdIha4naaBaaaleaaieGacqGFWaamaeqaaaaaaaa@3E2C@. (ii) In this study, the white noise n(t)=ddtω(t)
 MathType@MTEF@5@5@+=feaafiart1ev1aaatCvAUfKttLearuWrP9MDH5MBPbIqV92AaeXatLxBI9gBaebbnrfifHhDYfgasaacH8akY=wiFfYdH8Gipec8Eeeu0xXdbba9frFj0=OqFfea0dXdd9vqai=hGuQ8kuc9pgc9s8qqaq=dirpe0xb9q8qiLsFr0=vr0=vr0dc8meaabaqaciaacaGaaeqabaqabeGadaaakeaacqWGUbGBcqGGOaakcqWG0baDcqGGPaqkcqGH9aqpdaWcaaqaaiabdsgaKbqaaiabdsgaKjabdsha0baaiiGacqWFjpWDcqGGOaakcqWG0baDcqGGPaqkaaa@3B54@ is normalized with unit variance, in which the covariance of stochastic noise is absorbed by *M*.

Since the effect of intrinsic biochemical kinetic parametric fluctuation is state dependent and will influence the stability of genetic regulatory networks, it will be discussed first. Let *V*(*x*) > 0 with *V*(0) = 0 denote the Lyapunov (power like) function of a stochastic genetic regulatory network. In the linear genetic regulatory network, the Lyapunov function is always chosen as *V*(*x*) = *x*^*T*^*Px *for some symmetric positive definite matrix *P*. Then the equilibrium point *x *= 0 of a stochastic genetic regulatory network is stable in probability at the equilibrium point if the expectation of the derivative of *V*(*x*) is not positive [39], i.e., the total power (squares of concentrations) of the genetic regulatory network could not increase again in probability

E{ddtV(x)}≤0     (5)
 MathType@MTEF@5@5@+=feaafiart1ev1aaatCvAUfKttLearuWrP9MDH5MBPbIqV92AaeXatLxBI9gBaebbnrfifHhDYfgasaacH8akY=wiFfYdH8Gipec8Eeeu0xXdbba9frFj0=OqFfea0dXdd9vqai=hGuQ8kuc9pgc9s8qqaq=dirpe0xb9q8qiLsFr0=vr0=vr0dc8meaabaqaciaacaGaaeqabaqabeGadaaakeaacqWGfbqrdaGadaqaamaalaaabaGaemizaqgabaGaemizaqMaemiDaqhaaiabdAfawjabcIcaOiabdIha4jabcMcaPaGaay5Eaiaaw2haaiabgsMiJkabicdaWiaaxMaacaWLjaWaaeWaaeaacqaI1aqnaiaawIcacaGLPaaaaaa@3EDB@

Remark: In general, the nonlinear systems have many equilibrium points. If we are interested in the stability of the equilibrium point *x*_*e *_≠ 0, for the convenience of discussion [35], the origin should be shifted to the equilibrium point we are interested in, i.e., *x*^' ^= *x *- *x*_*e*_. Thus

d*x*'(*t*) = *N *(*x*' + *x*_*e*_)*dt *+ *M *(*x*' + *x*_*e*_) *dω *(*t*)     (6)

Then the stability problem of the equilibrium point *x*_*e *_in nonlinear stochastic network (4) is equivalent to the stability problem of *x*' = 0 in the nonlinear stochastic system (6).

In the linear stochastic genetic regulatory network (3), the robust stability under intrinsic fluctuation is given below

**Theorem 1: **The linear gene regulatory network with stochastic perturbation in equation (3) is stable in probability if the following Lyapunov-type equation

*PN *+ *N*^*T*^*P *+ *M*^*T*^*PM *≤ 0     (7)

has a symmetric positive definite solution *P *> 0.

**Proof: **See Appendix 1.

**Remark 1: **(i) In the intrinsic noise free case, i.e., the nominal case in equation (1), the stable condition is that the matrix inequality *PN *+ *N*^*T*^*P *≤ 0 has a symmetric positive definite solution *P *> 0. Obviously, the existence of a symmetric positive definite solution in (7) is more strict because the eigenvalues of system interaction matrix *N *should be located at the far left hand side of complex domain with large negative real values in order to overcome the extra term *M*^*T*^*PM *due to intrinsic noise in equation (7). If some eigenvalues of system interaction matrix *N *are near the *jω *axis, then these modes are more easily to perturb by intrinsic molecular fluctuation across the *jω *axis, such that the linear genetic regulatory network becomes unstable. Therefore, the smallest distance between the locations of the eigenvalues of *N *to *jω *axis can be considered as a robustness measure of linear genetic regulatory networks. (ii) It is easy to use LMI Toolbox of Matlab to find a positive definite *P *to solve (7) if it exists.

For the nonlinear stochastic regulatory network in equation (4), we obtain the robust stability theory under intrinsic parametric fluctuation around the equilibrium point *x*_*e *_= 0 as follows.

**Theorem 2: **The nonlinear perturbative genetic regulatory network in equation (4) is still stable in probability if the following Hamilton Jacobi inequality (HJI) has a positive solution *V*(*x*) > 0 with *V*(0) = 0

(∂V(x)∂x)TN(x)+12MT(x)∂2V(x)∂x2M(x)≤0     (8)
 MathType@MTEF@5@5@+=feaafiart1ev1aaatCvAUfKttLearuWrP9MDH5MBPbIqV92AaeXatLxBI9gBaebbnrfifHhDYfgasaacH8akY=wiFfYdH8Gipec8Eeeu0xXdbba9frFj0=OqFfea0dXdd9vqai=hGuQ8kuc9pgc9s8qqaq=dirpe0xb9q8qiLsFr0=vr0=vr0dc8meaabaqaciaacaGaaeqabaqabeGadaaakeaadaqadaqaamaalaaabaacciGae8NaIyRaemOvayLaeiikaGIaemiEaGNaeiykaKcabaGae8NaIyRaemiEaGhaaaGaayjkaiaawMcaamaaCaaaleqabaGaemivaqfaaOGaemOta4KaeiikaGIaemiEaGNaeiykaKIaey4kaSYaaSaaaeaacqaIXaqmaeaacqaIYaGmaaGaemyta00aaWbaaSqabeaacqWGubavaaGccqGGOaakcqWG4baEcqGGPaqkdaWcaaqaaiab=jGi2oaaCaaaleqabaGaeGOmaidaaOGaemOvayLaeiikaGIaemiEaGNaeiykaKcabaGae8NaIyRaemiEaG3aaWbaaSqabeaacqaIYaGmaaaaaOGaemyta0KaeiikaGIaemiEaGNaeiykaKIaeyizImQaeGimaaJaaCzcaiaaxMaadaqadaqaaiabiIda4aGaayjkaiaawMcaaaaa@5ADF@

i.e., the nonlinear stochastic regulatory network in equation (4) is stable in probability.

**Proof: **See Appendix 2.

**Remark 2: **(i) The inequality of equation (8) is an extension of inequality in equation (7) from the linear stochastic case to the nonlinear stochastic case. (ii) In general, it is not easy to solve the nonlinear inequality in equation (8) to see whether the stability of nonlinear stochastic genetic network is guaranteed under intrinsic fluctuation. A convenient method based on the so-called global linearization [38] is proposed in the following to solve the robust stability problem of nonlinear stochastic gene regulatory networks. Suppose the global linearization of *N*(*x*) is defined as

∂∂x[N(x)M(x)]∈Ω⊆R2n×n
 MathType@MTEF@5@5@+=feaafiart1ev1aaatCvAUfKttLearuWrP9MDH5MBPbIqV92AaeXatLxBI9gBaebbnrfifHhDYfgasaacH8akY=wiFfYdH8Gipec8Eeeu0xXdbba9frFj0=OqFfea0dXdd9vqai=hGuQ8kuc9pgc9s8qqaq=dirpe0xb9q8qiLsFr0=vr0=vr0dc8meaabaqaciaacaGaaeqabaqabeGadaaakeaadaWcaaqaaGGaciab=jGi2cqaaiab=jGi2kabdIha4baadaWadaqaauaabeqaceaaaeaacqWGobGtcqGGOaakcqWG4baEcqGGPaqkaeaacqWGnbqtcqGGOaakcqWG4baEcqGGPaqkaaaacaGLBbGaayzxaaGaeyicI4SaeuyQdCLaeyOHI0SaemOuai1aaWbaaSqabeaacqaIYaGmcqWGUbGBcqGHxdaTcqWGUbGBaaaaaa@47DC@ and suppose Ω is bounded by the following polytope with *n *vertices

Ω∈Co{[N1M1],[N2M2],…[NnMn]}     (9)
 MathType@MTEF@5@5@+=feaafiart1ev1aaatCvAUfKttLearuWrP9MDH5MBPbIqV92AaeXatLxBI9gBaebbnrfifHhDYfgasaacH8akY=wiFfYdH8Gipec8Eeeu0xXdbba9frFj0=OqFfea0dXdd9vqai=hGuQ8kuc9pgc9s8qqaq=dirpe0xb9q8qiLsFr0=vr0=vr0dc8meaabaqaciaacaGaaeqabaqabeGadaaakeaacqqHPoWvcqGHiiIZcqqGdbWqcqqGVbWBdaGadaqaamaadmaabaqbaeqabiqaaaqaaiabd6eaonaaBaaaleaacqaIXaqmaeqaaaGcbaGaemyta00aaSbaaSqaaiabigdaXaqabaaaaaGccaGLBbGaayzxaaGaeiilaWYaamWaaeaafaqabeGabaaabaGaemOta40aaSbaaSqaaiabikdaYaqabaaakeaacqWGnbqtdaWgaaWcbaGaeGOmaidabeaaaaaakiaawUfacaGLDbaacqGGSaalcqWIVlctdaWadaqaauaabeqaceaaaeaacqWGobGtdaWgaaWcbaGaemOBa4gabeaaaOqaaiabd2eannaaBaaaleaacqWGUbGBaeqaaaaaaOGaay5waiaaw2faaaGaay5Eaiaaw2haaiaaxMaacaWLjaWaaeWaaeaacqaI5aqoaiaawIcacaGLPaaaaaa@5083@

where Co{…} denotes the convex hull consisted by the vertices {…}. Therefore, all linearized systems of *dx*(*t*) = *N*(*x*)*dt *+ *M*(*x*)*dω*(*t*) are bounded in the polytope consisting of the linearized vertices *dx*(*t*) = *N*_*i*_*x(t)dt *+ M_*i*_*x(t)dω*(*t*), *i *= 1,2, … *L*. Then the perturbative nonlinear regulatory network is robustly stable under intrinsic noise if the following LMIs have a common positive solution *P *= *P*^*T *^> 0 [38]

PNi+NiTP+MiTPMi≤0for i=1,2,…,L     (10)
 MathType@MTEF@5@5@+=feaafiart1ev1aaatCvAUfKttLearuWrP9MDH5MBPbIqV92AaeXatLxBI9gBaebbnrfifHhDYfgasaacH8akY=wiFfYdH8Gipec8Eeeu0xXdbba9frFj0=OqFfea0dXdd9vqai=hGuQ8kuc9pgc9s8qqaq=dirpe0xb9q8qiLsFr0=vr0=vr0dc8meaabaqaciaacaGaaeqabaqabeGadaaakeaafaqabeqacaaabaGaemiuaaLaemOta40aaSbaaSqaaiabdMgaPbqabaGccqGHRaWkcqWGobGtdaqhaaWcbaGaemyAaKgabaGaemivaqfaaOGaemiuaaLaey4kaSIaemyta00aa0baaSqaaiabdMgaPbqaaiabdsfaubaakiabdcfaqjabd2eannaaBaaaleaacqWGPbqAaeqaaOGaeyizImQaeGimaadabaGaeeOzayMaee4Ba8MaeeOCaiNaeeiiaaIaemyAaKMaeyypa0JaeGymaeJaeiilaWIaeGOmaiJaeiilaWIaeS47IWKaemitaWeaaiaaxMaacaWLjaWaaeWaaeaacqaIXaqmcqaIWaamaiaawIcacaGLPaaaaaa@547A@

In general, it is very easy to find a common *P *> 0 to solve the above LMIs by the LMI Toolbox in Matlab if it exists. Therefore, it is more appealing to solve a *P *> 0 of LMIs in (10) than to solve a *V*(*x*) > 0 of HJI in equation (8) to guarantee the robust stability of nonlinear stochastic gene regulatory network under intrinsic noise, but the results of the LMI method may be more conservative.

#### Attenuation and amplification of extrinsic noise

After the robust stability of genetic regulatory network is guaranteed under the intrinsic biochemical parametric fluctuation, the effect of the extrinsic fluctuation on the network will be discussed. If the linear regulatory network in equation (2) also suffers the extrinsic signals *υ*(*t*) (or noises, see Figure 1) outside the network, then stochastic equation (2) is modified as follows

dx(t)=(Nx(t)+Hυ(t))dt+Mx(t)dω(t)Z(t)=Cx(t)     (11)
 MathType@MTEF@5@5@+=feaafiart1ev1aaatCvAUfKttLearuWrP9MDH5MBPbIqV92AaeXatLxBI9gBaebbnrfifHhDYfgasaacH8akY=wiFfYdH8Gipec8Eeeu0xXdbba9frFj0=OqFfea0dXdd9vqai=hGuQ8kuc9pgc9s8qqaq=dirpe0xb9q8qiLsFr0=vr0=vr0dc8meaabaqaciaacaGaaeqabaqabeGadaaakeaafaqaaeGabaaabaGaemizaqMaemiEaGNaeiikaGIaemiDaqNaeiykaKIaeyypa0ZaaeWaaeaacqWGobGtcqWG4baEcqGGOaakcqWG0baDcqGGPaqkcqGHRaWkcqWGibasiiGacqWFfpqDcqGGOaakcqWG0baDcqGGPaqkaiaawIcacaGLPaaacqWGKbazcqWG0baDcqGHRaWkcqWGnbqtcqWG4baEcqGGOaakcqWG0baDcqGGPaqkcqWGKbazcqWFjpWDcqGGOaakcqWG0baDcqGGPaqkaeaacqWGAbGwcqGGOaakcqWG0baDcqGGPaqkcqGH9aqpcqWGdbWqcqWG4baEcqGGOaakcqWG0baDcqGGPaqkaaGaaCzcaiaaxMaadaqadaqaaiabigdaXiabigdaXaGaayjkaiaawMcaaaaa@613E@

where *H *is a coupling matrix which denotes the influence of extrinsic signal on the state *x*(*t*). *Z*(*t*) denotes the concentration of some genes that we are interested in. For example, if we only want to discuss the effect of noises of *ω*(*t*) and *υ*(*t*) on gene *i*, i.e., *x*_*i*_(*t*), then we let *C *= [000…1…00] i.e., every element of *C *is zero except 1 at the *i*th element. If we want to discuss the effect of noises on the whole genetic regulatory network, then we let *C *= *I*, the identity matrix.

Similarly, the nonlinear stochastic regulatory networks in equation (4) under extrinsic fluctuation should be modified as

dx(t)=(N(x)+H(x)υ(t))dt+M(x)dω(t)Z(t)=Cx(t)     (12)
 MathType@MTEF@5@5@+=feaafiart1ev1aaatCvAUfKttLearuWrP9MDH5MBPbIqV92AaeXatLxBI9gBaebbnrfifHhDYfgasaacH8akY=wiFfYdH8Gipec8Eeeu0xXdbba9frFj0=OqFfea0dXdd9vqai=hGuQ8kuc9pgc9s8qqaq=dirpe0xb9q8qiLsFr0=vr0=vr0dc8meaabaqaciaacaGaaeqabaqabeGadaaakeaafaqaaeGabaaabaGaemizaqMaemiEaGNaeiikaGIaemiDaqNaeiykaKIaeyypa0ZaaeWaaeaacqWGobGtcqGGOaakcqWG4baEcqGGPaqkcqGHRaWkcqWGibascqGGOaakcqWG4baEcqGGPaqkiiGacqWFfpqDcqGGOaakcqWG0baDcqGGPaqkaiaawIcacaGLPaaacqWGKbazcqWG0baDcqGHRaWkcqWGnbqtcqGGOaakcqWG4baEcqGGPaqkcqWGKbazcqWFjpWDcqGGOaakcqWG0baDcqGGPaqkaeaacqWGAbGwcqGGOaakcqWG0baDcqGGPaqkcqGH9aqpcqWGdbWqcqWG4baEcqGGOaakcqWG0baDcqGGPaqkaaGaaCzcaiaaxMaadaqadaqaaiabigdaXiabikdaYaGaayjkaiaawMcaaaaa@6189@

The attenuation and amplification of extrinsic noise of stochastic regulatory networks in equations (11) and (12) will be discussed in the following theorems.

Let us denote L_2_-norm of *υ*(*t*) as

‖υ(t)‖2={E{∫0∞υ(t)Tυ(t)dt}}12     (13)
 MathType@MTEF@5@5@+=feaafiart1ev1aaatCvAUfKttLearuWrP9MDH5MBPbIqV92AaeXatLxBI9gBaebbnrfifHhDYfgasaacH8akY=wiFfYdH8Gipec8Eeeu0xXdbba9frFj0=OqFfea0dXdd9vqai=hGuQ8kuc9pgc9s8qqaq=dirpe0xb9q8qiLsFr0=vr0=vr0dc8meaabaqaciaacaGaaeqabaqabeGadaaakeaadaqbdaqaaGGaciab=v8a1jabcIcaOiabdsha0jabcMcaPaGaayzcSlaawQa7amaaBaaaleaacqaIYaGmaeqaaOGaeyypa0ZaaiWaaeaacqWGfbqrdaGadaqaamaapedabaGae8xXduNaeiikaGIaemiDaqNaeiykaKYaaWbaaSqabeaacqWGubavaaGccqWFfpqDcqGGOaakcqWG0baDcqGGPaqkcqWGKbazcqWG0baDaSqaaiabicdaWaqaaiabg6HiLcqdcqGHRiI8aaGccaGL7bGaayzFaaaacaGL7bGaayzFaaWaaWbaaSqabeaadaWcaaqaaiabigdaXaqaaiabikdaYaaaaaGccaWLjaGaaCzcamaabmaabaGaeGymaeJaeG4mamdacaGLOaGaayzkaaaaaa@55C7@

where *E *denotes the expectation. We denote *υ*(*t*) ∈ L_2_, if || *υ*(*t*)|| _2 _< ∞ Then the positive value *ρ *in the following inequality is called the effect (or gain) from the extrinsic noise *υ*(*t*) to *Z(t) *in the perturbative genetic regulatory network with *x*(0) = 0 [34,36,38]

‖Z(t)‖2‖υ(t)‖2≤ρor‖Z(t)‖2≤ρ‖υ(t)‖2,∀υ(t)∈L2     (14)
 MathType@MTEF@5@5@+=feaafiart1ev1aaatCvAUfKttLearuWrP9MDH5MBPbIqV92AaeXatLxBI9gBaebbnrfifHhDYfgasaacH8akY=wiFfYdH8Gipec8Eeeu0xXdbba9frFj0=OqFfea0dXdd9vqai=hGuQ8kuc9pgc9s8qqaq=dirpe0xb9q8qiLsFr0=vr0=vr0dc8meaabaqaciaacaGaaeqabaqabeGadaaakeaafaqabeqaeaaaaeaadaWcaaqaamaafmaabaGaemOwaOLaeiikaGIaemiDaqNaeiykaKcacaGLjWUaayPcSdWaaSbaaSqaaiabikdaYaqabaaakeaadaqbdaqaaGGaciab=v8a1jabcIcaOiabdsha0jabcMcaPaGaayzcSlaawQa7amaaBaaaleaacqaIYaGmaeqaaaaakiabgsMiJkab=f8aYbqaaiabb+gaVjabbkhaYbqaamaafmaabaGaemOwaOLaeiikaGIaemiDaqNaeiykaKcacaGLjWUaayPcSdWaaSbaaSqaaiabikdaYaqabaGccqGHKjYOcqWFbpGCdaqbdaqaaiab=v8a1Hqaaiab+HcaOiab+rha0jab+LcaPaGaayzcSlaawQa7amaaBaaaleaacqaIYaGmaeqaaOGaeiilaWcabaGaeyiaIiIae8xXduNae4hkaGIae4hDaqNae4xkaKIaeyicI4SaeeitaW0aaSbaaSqaaiabbkdaYaqabaaaaOGaaCzcaiaaxMaadaqadaqaaiabigdaXiabisda0aGaayjkaiaawMcaaaaa@694A@

If *ρ *< 1, we say the extrinsic noise *υ*(*t*) is attenuated at *Z(t) *by the genetic regulatory network. If *ρ *> 1, we say the extrinsic noise *υ*(*t*) is amplified at *Z*(*t*) by the genetic regulatory network. In this situation, *ρ *is called the attenuation level if *ρ *< 1 or amplification level if *ρ *> 1. If *ρ *= 1, we call it lossless. In the inequality (14), it is under the assumption of *x*(0) = 0, i.e., all signals in the gene network are driven by noises. If the initial condition is not zero, i.e., *x*(0) ≠ 0, then an extra term of initial condition should be added as follows [15,36]

‖Z(t)‖22≤E{V(x(0))}+ρ2‖υ(t)‖22     (15)
 MathType@MTEF@5@5@+=feaafiart1ev1aaatCvAUfKttLearuWrP9MDH5MBPbIqV92AaeXatLxBI9gBaebbnrfifHhDYfgasaacH8akY=wiFfYdH8Gipec8Eeeu0xXdbba9frFj0=OqFfea0dXdd9vqai=hGuQ8kuc9pgc9s8qqaq=dirpe0xb9q8qiLsFr0=vr0=vr0dc8meaabaqaciaacaGaaeqabaqabeGadaaakeaadaqbdaqaaiabdQfaAjabcIcaOiabdsha0jabcMcaPaGaayzcSlaawQa7amaaDaaaleaacqaIYaGmaeaacqaIYaGmaaGccqGHKjYOcqWGfbqrdaGadaqaaiabdAfawjabcIcaOiabdIha4jabcIcaOiabicdaWiabcMcaPiabcMcaPaGaay5Eaiaaw2haaiabgUcaRGGaciab=f8aYnaaCaaaleqabaGaeGOmaidaaOWaauWaaeaacqWFfpqDcqGGOaakcqWG0baDcqGGPaqkaiaawMa7caGLkWoadaqhaaWcbaGaeGOmaidabaGaeGOmaidaaOGaaCzcaiaaxMaadaqadaqaaiabigdaXiabiwda1aGaayjkaiaawMcaaaaa@54F5@

for some positive function *V*(*x*(0)) of the initial condition *x*(0) .

Remark: If *υ*(*t*) is a deterministic signal from the environment, then ‖υ(t)‖2={E{∫0∞υ(t)Tυ(t)dt}}12
 MathType@MTEF@5@5@+=feaafiart1ev1aaatCvAUfKttLearuWrP9MDH5MBPbIqV92AaeXatLxBI9gBaebbnrfifHhDYfgasaacH8akY=wiFfYdH8Gipec8Eeeu0xXdbba9frFj0=OqFfea0dXdd9vqai=hGuQ8kuc9pgc9s8qqaq=dirpe0xb9q8qiLsFr0=vr0=vr0dc8meaabaqaciaacaGaaeqabaqabeGadaaakeaadaqbdaqaaGGaciab=v8a1jabcIcaOiabdsha0jabcMcaPaGaayzcSlaawQa7amaaBaaaleaacqaIYaGmaeqaaOGaeyypa0ZaaiWaaeaacqWGfbqrdaGadaqaamaapedabaGae8xXduNaeiikaGIaemiDaqNaeiykaKYaaWbaaSqabeaacqWGubavaaGccqWFfpqDcqGGOaakcqWG0baDcqGGPaqkcqWGKbazcqWG0baDaSqaaiabicdaWaqaaiabg6HiLcqdcqGHRiI8aaGccaGL7bGaayzFaaaacaGL7bGaayzFaaWaaWbaaSqabeaadaWcaaqaaiabigdaXaqaaiabikdaYaaaaaaaaa@510C@ should be changed to ‖υ(t)‖2={∫0∞υ(t)Tυ(t)dt}12
 MathType@MTEF@5@5@+=feaafiart1ev1aaatCvAUfKttLearuWrP9MDH5MBPbIqV92AaeXatLxBI9gBaebbnrfifHhDYfgasaacH8akY=wiFfYdH8Gipec8Eeeu0xXdbba9frFj0=OqFfea0dXdd9vqai=hGuQ8kuc9pgc9s8qqaq=dirpe0xb9q8qiLsFr0=vr0=vr0dc8meaabaqaciaacaGaaeqabaqabeGadaaakeaadaqbdaqaaGGaciab=v8a1jabcIcaOiabdsha0jabcMcaPaGaayzcSlaawQa7amaaBaaaleaacqaIYaGmaeqaaOGaeyypa0ZaaiWaaeaadaWdXaqaaiab=v8a1jabcIcaOiabdsha0jabcMcaPmaaCaaaleqabaGaemivaqfaaOGae8xXduNaeiikaGIaemiDaqNaeiykaKIaemizaqMaemiDaqhaleaacqaIWaamaeaacqGHEisPa0Gaey4kIipaaOGaay5Eaiaaw2haamaaCaaaleqabaWaaSaaaeaacqaIXaqmaeaacqaIYaGmaaaaaaaa@4DC8@ in (14) and (15).

Based on the analysis above, some theorems about the attenuation or amplification of extrinsic noise of stochastic genetic regulatory networks are discussed below

**Theorem 3: **The attenuation level *ρ *of linear perturbative genetic regulatory network in equation (11) is guaranteed if the following inequality has a positive definite solution *P > *0

PN+NTP+MTPM+CTC+1ρ2PHHTP≤0     (16)
 MathType@MTEF@5@5@+=feaafiart1ev1aaatCvAUfKttLearuWrP9MDH5MBPbIqV92AaeXatLxBI9gBaebbnrfifHhDYfgasaacH8akY=wiFfYdH8Gipec8Eeeu0xXdbba9frFj0=OqFfea0dXdd9vqai=hGuQ8kuc9pgc9s8qqaq=dirpe0xb9q8qiLsFr0=vr0=vr0dc8meaabaqaciaacaGaaeqabaqabeGadaaakeaacqWGqbaucqWGobGtcqGHRaWkcqWGobGtdaahaaWcbeqaaiabdsfaubaakiabdcfaqjabgUcaRiabd2eannaaCaaaleqabaGaemivaqfaaOGaemiuaaLaemyta0Kaey4kaSIaem4qam0aaWbaaSqabeaacqWGubavaaGccqWGdbWqcqGHRaWkdaWcaaqaaiabigdaXaqaaGGaciab=f8aYnaaCaaaleqabaGaeGOmaidaaaaakiabdcfaqjabdIeaijabdIeainaaCaaaleqabaGaemivaqfaaOGaemiuaaLaeyizImQaeGimaaJaaCzcaiaaxMaadaqadaqaaiabigdaXiabiAda2aGaayjkaiaawMcaaaaa@4FCB@

By Shur complement [38], inequality (16) is equivalent to the following LMI

[PN+NTP+CTC+MTPMPH(PH)T−ρ2I]≤0     (17)
 MathType@MTEF@5@5@+=feaafiart1ev1aaatCvAUfKttLearuWrP9MDH5MBPbIqV92AaeXatLxBI9gBaebbnrfifHhDYfgasaacH8akY=wiFfYdH8Gipec8Eeeu0xXdbba9frFj0=OqFfea0dXdd9vqai=hGuQ8kuc9pgc9s8qqaq=dirpe0xb9q8qiLsFr0=vr0=vr0dc8meaabaqaciaacaGaaeqabaqabeGadaaakeaadaWadaqaauaabeqaciaaaeaacqWGqbaucqWGobGtcqGHRaWkcqWGobGtdaahaaWcbeqaaiabdsfaubaakiabdcfaqjabgUcaRiabdoeadnaaCaaaleqabaGaemivaqfaaOGaem4qamKaey4kaSIaemyta00aaWbaaSqabeaacqWGubavaaGccqWGqbaucqWGnbqtaeaacqWGqbaucqWGibasaeaacqGGOaakcqWGqbaucqWGibascqGGPaqkdaahaaWcbeqaaiabdsfaubaaaOqaaiabgkHiTGGaciab=f8aYnaaCaaaleqabaGaeGOmaidaaOGaemysaKeaaaGaay5waiaaw2faaiabgsMiJkabicdaWiaaxMaacaWLjaWaaeWaaeaacqaIXaqmcqaI3aWnaiaawIcacaGLPaaaaaa@53A7@

**Proof: **See Appendix 3.

The optimal attenuation level *ρ*_*o *_of the linear stochastic genetic regulatory network (11) can be obtained by solving the following constrained optimization

ρo=min⁡ρsubject toρ>0,P>0 and (17)     (18)
 MathType@MTEF@5@5@+=feaafiart1ev1aaatCvAUfKttLearuWrP9MDH5MBPbIqV92AaeXatLxBI9gBaebbnrfifHhDYfgasaacH8akY=wiFfYdH8Gipec8Eeeu0xXdbba9frFj0=OqFfea0dXdd9vqai=hGuQ8kuc9pgc9s8qqaq=dirpe0xb9q8qiLsFr0=vr0=vr0dc8meaabaqaciaacaGaaeqabaqabeGadaaakeaafaqaaeGabaaabaacciGae8xWdi3aaSbaaSqaaGqaciab+9gaVbqabaGccqGH9aqpcyGGTbqBcqGGPbqAcqGGUbGBcqWFbpGCaeaacqqGZbWCcqqG1bqDcqqGIbGycqqGQbGAcqqGLbqzcqqGJbWycqqG0baDcqqGGaaicqqG0baDcqqGVbWBtCvAUfeBSjuyZL2yd9gzLbvyNv2CaeHbwvMCKfMBHbaceaGaa0hiaiab=f8aYjab=5da+GGaaiab8bdaWiab8XcaSiab+bfaqHqaaiab75da+iab7bdaWiabbccaGiabbggaHjabb6gaUjabbsgaKjabbccaGiab7HcaOiab7fdaXiab7Dda3iab7LcaPaaacaWLjaGaaCzcamaabmaabaGaeGymaeJaeGioaGdacaGLOaGaayzkaaaaaa@64A2@

The above LMI optimization can be solved easily by decreasing *ρ *until no positive definite solution *P *> 0 for equation (17) can be found. Software packages such as LMI Optimization Toolbox in Matlab have been developed to easily solve the above LMI optimization.

**Remark 3: **(i) If *ρ*_*o *_< 1 in equation (18), the extrinsic noise *υ*(*t*) is attenuated by the genetic regulatory network at *Z*(*t*) i.e., this gene is less sensitive to the environmental noise. (ii) If *ρ*_*o *_> 1, the extrinsic noise *υ*(*t*) is amplified by the genetic regulatory network at *Z*(*t*) i.e., this gene is more sensitive to the environmental noise. (iii) If we only want to check if the genetic regulatory network has a prescribed attenuation level *ρ*, we just check if inequality (17) has a positive definite solution *P *> 0.

For the nonlinear perturbative genetic regulatory network in equation (12), the attenuation or amplification of extrinsic noise *υ*(*t*) at *Z*(*t*) is discussed in the following theorem.

**Theorem 4: **The attenuation level *ρ *of nonlinear genetic regulatory network (12) is guaranteed if the following Hamilton-Jacobi inequality has a positive definite solution V(x)>0xTCTCx+(∂V(x)∂x)TN(x)+12MT(x)∂2V(x)∂x2M(x)+14ρ2(∂V(x)∂x)THHT∂V(x)∂x≤0     (19)
 MathType@MTEF@5@5@+=feaafiart1ev1aaatCvAUfKttLearuWrP9MDH5MBPbIqV92AaeXatLxBI9gBaebbnrfifHhDYfgasaacH8akY=wiFfYdH8Gipec8Eeeu0xXdbba9frFj0=OqFfea0dXdd9vqai=hGuQ8kuc9pgc9s8qqaq=dirpe0xb9q8qiLsFr0=vr0=vr0dc8meaabaqaciaacaGaaeqabaqabeGadaaakeaafaqaaeGabaaabaGaemOvayLaeiikaGIaemiEaGNaeiykaKIaeyOpa4JaeGimaadabaGaemiEaG3aaWbaaSqabeaacqWGubavaaGccqWGdbWqdaahaaWcbeqaaiabdsfaubaakiabdoeadjabdIha4jabgUcaRmaabmaabaWaaSaaaeaaiiGacqWFciITcqWGwbGvcqGGOaakcqWG4baEcqGGPaqkaeaacqWFciITcqWG4baEaaaacaGLOaGaayzkaaWaaWbaaSqabeaacqWGubavaaGccqWGobGtcqGGOaakcqWG4baEcqGGPaqkcqGHRaWkdaWcaaqaaiabigdaXaqaaiabikdaYaaacqWGnbqtdaahaaWcbeqaaiabdsfaubaakiabcIcaOiabdIha4jabcMcaPmaalaaabaGae8NaIy7aaWbaaSqabeaacqaIYaGmaaGccqWGwbGvcqGGOaakcqWG4baEcqGGPaqkaeaacqWFciITcqWG4baEdaahaaWcbeqaaiabikdaYaaaaaGccqWGnbqtcqGGOaakcqWG4baEcqGGPaqkcqGHRaWkdaWcaaqaaiabigdaXaqaaiabisda0iab=f8aYnaaCaaaleqabaGaeGOmaidaaaaakmaabmaabaWaaSaaaeaacqWFciITcqWGwbGvcqGGOaakcqWG4baEcqGGPaqkaeaacqWFciITcqWG4baEaaaacaGLOaGaayzkaaWaaWbaaSqabeaacqWGubavaaGccqWGibascqWGibasdaahaaWcbeqaaiabdsfaubaakmaalaaabaGae8NaIyRaemOvayLaeiikaGIaemiEaGNaeiykaKcabaGae8NaIyRaemiEaGhaaiabgsMiJkabicdaWiaaxMaacaWLjaWaaeWaaeaacqaIXaqmcqaI5aqoaiaawIcacaGLPaaaaaaaaa@888A@

**Proof: **See Appendix 4.

The optimal attenuation level *ρ*_*o *_of the nonlinear stochastic regulatory network can be obtained by solving the following constrained optimization

ρ0=min⁡V(x) ρsubject to ρ>0,P>0 and (19)     (20)
 MathType@MTEF@5@5@+=feaafiart1ev1aaatCvAUfKttLearuWrP9MDH5MBPbIqV92AaeXatLxBI9gBaebbnrfifHhDYfgasaacH8akY=wiFfYdH8Gipec8Eeeu0xXdbba9frFj0=OqFfea0dXdd9vqai=hGuQ8kuc9pgc9s8qqaq=dirpe0xb9q8qiLsFr0=vr0=vr0dc8meaabaqaciaacaGaaeqabaqabeGadaaakeaafaqaaeGabaaabaacciGae8xWdi3aaSbaaSqaaGqaciab+bdaWaqabaGccqGH9aqpdaWfqaqaaiGbc2gaTjabcMgaPjabc6gaUbWcbaGaemOvayLaeiikaGIaemiEaGNaeiykaKcabeaakiabbccaGiab=f8aYbqaaiabbohaZjabbwha1jabbkgaIjabbQgaQjabbwgaLjabbogaJjabbsha0jabbccaGiabbsha0jabb+gaVjabbccaGiab=f8aYjabg6da+iabicdaWiabcYcaSiabdcfaqjabg6da+iabicdaWiabbccaGiabbggaHjabb6gaUjabbsgaKjabbccaGiabbIcaOiabbgdaXiabbMda5iabbMcaPaaacaWLjaGaaCzcamaabmaabaGaeGOmaiJaeGimaadacaGLOaGaayzkaaaaaa@5F5C@

**Remark 4: **(i) In general, there exists no systematic method to solve the constrained optimizations in (20). It should be solved case by case by decreasing *ρ *until no positive solution *V*(*x*) for the Hamilton-Jacobi inequality in (19) can be found. (ii) An approximation solution for (20) based on the "global linearization" techniques of (9) and (10) in Remark 2 (ii) is introduced in the following. Thus, if the nonlinear perturbative genetic regulatory network (12) could be bounded in the polytope consisting of *L *linearized vertices [38]* dx(t) *= (*N*_*i*_*x*(*t*) + *H*_*i*_*υ*(*t*))*dt *+ *M*_*i*_*x*(*t*)*dω*(*t*), *i *= 1,2, …*L*, then after some rearrangements the optimal attenuation level *ρ*_*o *_in Theorem 4 can be approximated by solving the following constrained optimization problem

ρo=min ⁡Pρsubject toρ>0, P>0,[PNi+NiTP+MiTPMi+CTCPHi(PHi)T−ρ2I]≤0,for i=1,2,…,L     (21)
 MathType@MTEF@5@5@+=feaafiart1ev1aaatCvAUfKttLearuWrP9MDH5MBPbIqV92AaeXatLxBI9gBaebbnrfifHhDYfgasaacH8akY=wiFfYdH8Gipec8Eeeu0xXdbba9frFj0=OqFfea0dXdd9vqai=hGuQ8kuc9pgc9s8qqaq=dirpe0xb9q8qiLsFr0=vr0=vr0dc8meaabaqaciaacaGaaeqabaqabeGadaaakeaafaqaaeabbaaaaeaaiiGacqWFbpGCdaWgaaWcbaacbiGae4hmaadabeaakiabg2da9maaxababaGagiyBa0MaeiyAaKMaeiOBa4galeaacqWGqbauaeqaaOGae8xWdihabaGaee4CamNaeeyDauNaeeOyaiMaeeOAaOMaeeyzauMaee4yamMaeeiDaqNaeeiiaaIaeeiDaqNaee4Ba8gabaGae8xWdiNaeyOpa4JaeGimaaJaeiilaWIaeeiiaaIaemiuaaLaeyOpa4JaeGimaaJaeiilaWcabaqbaeqabeGaaaqaamaadmaabaqbaeqabiGaaaqaaiabdcfaqjabd6eaonaaBaaaleaacqWGPbqAaeqaaOGaey4kaSIaemOta40aa0baaSqaaiabdMgaPbqaaiabdsfaubaakiabdcfaqjabgUcaRiabd2eannaaDaaaleaacqWGPbqAaeaacqWGubavaaGccqWGqbaucqWGnbqtdaWgaaWcbaGaemyAaKgabeaakiabgUcaRiabdoeadnaaCaaaleqabaGaemivaqfaaOGaem4qameabaGaemiuaaLaemisaG0aaSbaaSqaaiabdMgaPbqabaaakeaacqGGOaakcqWGqbaucqWGibasdaWgaaWcbaGaemyAaKgabeaakiabcMcaPmaaCaaaleqabaGaemivaqfaaaGcbaGaeyOeI0Iae8xWdi3aaWbaaSqabeaacqaIYaGmaaGccqWGjbqsaaaacaGLBbGaayzxaaGaeyizImQaeGimaaJaeiilaWcabaGaeeOzayMaee4Ba8MaeeOCaiNaeeiiaaIaemyAaKMaeyypa0JaeGymaeJaeiilaWIaeGOmaiJaeiilaWIaeS47IWKaeiilaWIaemitaWeaaaaacaWLjaGaaCzcamaabmaabaGaeGOmaiJaeGymaedacaGLOaGaayzkaaaaaa@8E22@

This result is similar to the constrained optimization in (18) except that a set of LMI constraints, i.e., the HJI constraint in (19) is replaced by a set of LMI constraints in (21) to make the solution feasible. However, it may lead to a conservative result.

### Computational simulations

To confirm the validity of the stability robustness and the noise attenuation or amplification schemes we proposed, we conducted several computational simulations of a typical genetic regulatory network. The detailed equations and parameters are shown in Appendix 5.

**Figure 2:**
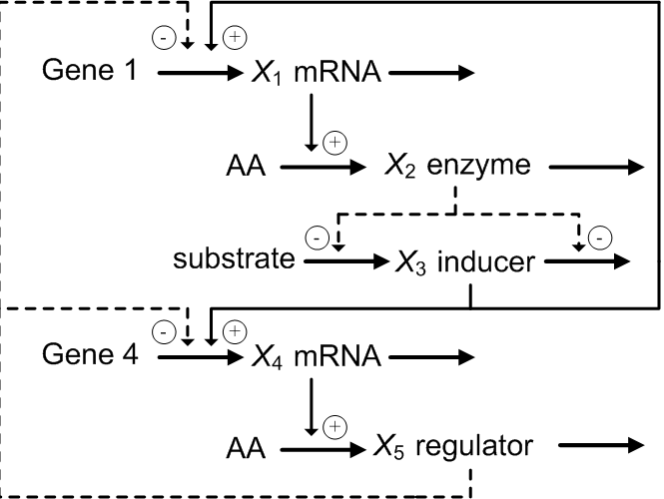
A typical genetic regulatory network describing the gene, mRNA and protein interactions [40].

Consider a typical genetic regulatory network, as shown in Figure 2 [40,41]. This is a typical gene interaction system describing the gene, mRNA and protein interactions. *X*_*1 *_is an mRNA produced from gene 1, *X*_2 _is an enzyme protein that it produces, and *X*_3 _is an inducer protein catalyzed by *X*_2_. In addition, *X*_4 _is an mRNA produced from gene 4 and *X*_5 _is a regulator protein it produces. Positive feedback from the inducer protein *X*_3 _and negative feedback from the regulator protein *X*_5 _are assumed in the mRNA production processes of gene 1 and gene 4 [42]. The genetic regulatory network can be represented as follows

X˙1=k1X3X5−1−k2X12X1(0)=0.5X˙2=k3X12−k4X22X2(0)=0.5X˙3=k5X2−1−k6X2−1X32X3(0)=0.5X˙4=k7X32X5−1−k8X42X4(0)=0.5X˙5=k9X42−k10X52X5(0)=0.5     (22)
 MathType@MTEF@5@5@+=feaafiart1ev1aaatCvAUfKttLearuWrP9MDH5MBPbIqV92AaeXatLxBI9gBaebbnrfifHhDYfgasaacH8akY=wiFfYdH8Gipec8Eeeu0xXdbba9frFj0=OqFfea0dXdd9vqai=hGuQ8kuc9pgc9s8qqaq=dirpe0xb9q8qiLsFr0=vr0=vr0dc8meaabaqaciaacaGaaeqabaqabeGadaaakeaafaqaaeqbcaaaaeaacuWGybawgaGaamaaBaaaleaacqaIXaqmaeqaaOGaeyypa0Jaem4AaS2aaSbaaSqaaiabigdaXaqabaGccqWGybawdaWgaaWcbaGaeG4mamdabeaakiabdIfaynaaDaaaleaacqaI1aqnaeaacqGHsislcqaIXaqmaaGccqGHsislcqWGRbWAdaWgaaWcbaGaeGOmaidabeaakiabdIfaynaaDaaaleaacqaIXaqmaeaacqaIYaGmaaaakeaacqWGybawdaWgaaWcbaGaeGymaedabeaakiabcIcaOiabicdaWiabcMcaPiabg2da9iabicdaWiabc6caUiabiwda1aqaaiqbdIfayzaacaWaaSbaaSqaaiabikdaYaqabaGccqGH9aqpcqWGRbWAdaWgaaWcbaGaeG4mamdabeaakiabdIfaynaaDaaaleaacqaIXaqmaeaacqaIYaGmaaGccqGHsislcqWGRbWAdaWgaaWcbaGaeGinaqdabeaakiabdIfaynaaDaaaleaacqaIYaGmaeaacqaIYaGmaaaakeaacqWGybawdaWgaaWcbaGaeGOmaidabeaakiabcIcaOiabicdaWiabcMcaPiabg2da9iabicdaWiabc6caUiabiwda1aqaaiqbdIfayzaacaWaaSbaaSqaaiabiodaZaqabaGccqGH9aqpcqWGRbWAdaWgaaWcbaGaeGynaudabeaakiabdIfaynaaDaaaleaacqaIYaGmaeaacqGHsislcqaIXaqmaaGccqGHsislcqWGRbWAdaWgaaWcbaGaeGOnaydabeaakiabdIfaynaaDaaaleaacqaIYaGmaeaacqGHsislcqaIXaqmaaGccqWGybawdaqhaaWcbaGaeG4mamdabaGaeGOmaidaaaGcbaGaemiwaG1aaSbaaSqaaiabiodaZaqabaGccqGGOaakcqaIWaamcqGGPaqkcqGH9aqpcqaIWaamcqGGUaGlcqaI1aqnaeaacuWGybawgaGaamaaBaaaleaacqaI0aanaeqaaOGaeyypa0Jaem4AaS2aaSbaaSqaaiabiEda3aqabaGccqWGybawdaqhaaWcbaGaeG4mamdabaGaeGOmaidaaOGaemiwaG1aa0baaSqaaiabiwda1aqaaiabgkHiTiabigdaXaaakiabgkHiTiabdUgaRnaaBaaaleaacqaI4aaoaeqaaOGaemiwaG1aa0baaSqaaiabisda0aqaaiabikdaYaaaaOqaaiabdIfaynaaBaaaleaacqaI0aanaeqaaOGaeiikaGIaeGimaaJaeiykaKIaeyypa0JaeGimaaJaeiOla4IaeGynaudabaGafmiwaGLbaiaadaWgaaWcbaGaeGynaudabeaakiabg2da9iabdUgaRnaaBaaaleaacqaI5aqoaeqaaOGaemiwaG1aa0baaSqaaiabisda0aqaaiabikdaYaaakiabgkHiTiabdUgaRnaaBaaaleaacqaIXaqmcqaIWaamaeqaaOGaemiwaG1aa0baaSqaaiabiwda1aqaaiabikdaYaaaaOqaaiabdIfaynaaBaaaleaacqaI1aqnaeqaaOGaeiikaGIaeGimaaJaeiykaKIaeyypa0JaeGimaaJaeiOla4IaeGynaudaaiaaxMaacaWLjaWaaeWaaeaacqaIYaGmcqaIYaGmaiaawIcacaGLPaaaaaa@BBF0@

In the nominal case, the kinetic parameters are

**Figure 3:**
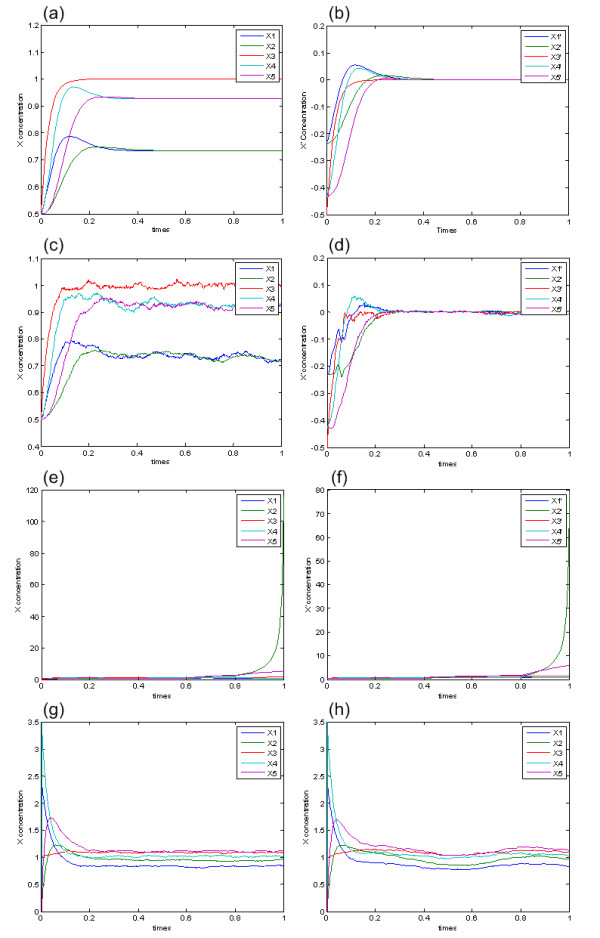
**The dynamic responses of the genetic regulatory network in computational simulation under different stochastic perturbative cases**. (a) The case without perturbation. (b) The case without perturbation but under coordinate shift. (c) The case with perturbation in (A18). (d) The case with perturbation and under coordinate shift in (A19). (e) The case with perturbation in (A20). (f) The case with perturbation and under coordinate shift in (A21). (g) The noise-driven case in (A22). (h) The signal-driven case in (A22) when *υ*(t) = 2+sin(10t).

k1=5k2=10k3=10k4=10k5=10k6=10k7=8k8=10k9=10k10=10
 MathType@MTEF@5@5@+=feaafiart1ev1aaatCvAUfKttLearuWrP9MDH5MBPbIqV92AaeXatLxBI9gBaebbnrfifHhDYfgasaacH8akY=wiFfYdH8Gipec8Eeeu0xXdbba9frFj0=OqFfea0dXdd9vqai=hGuQ8kuc9pgc9s8qqaq=dirpe0xb9q8qiLsFr0=vr0=vr0dc8meaabaqaciaacaGaaeqabaqabeGadaaakeaafaqaaeqbcaaaaeaacqWGRbWAdaWgaaWcbaGaeGymaedabeaakiabg2da9iabiwda1aqaaiabdUgaRnaaBaaaleaacqaIYaGmaeqaaOGaeyypa0JaeGymaeJaeGimaadabaGaem4AaS2aaSbaaSqaaiabiodaZaqabaGccqGH9aqpcqaIXaqmcqaIWaamaeaacqWGRbWAdaWgaaWcbaGaeGinaqdabeaakiabg2da9iabigdaXiabicdaWaqaaiabdUgaRnaaBaaaleaacqaI1aqnaeqaaOGaeyypa0JaeGymaeJaeGimaadabaGaem4AaS2aaSbaaSqaaiabiAda2aqabaGccqGH9aqpcqaIXaqmcqaIWaamaeaacqWGRbWAdaWgaaWcbaGaeG4naCdabeaakiabg2da9iabiIda4aqaaiabdUgaRnaaBaaaleaacqaI4aaoaeqaaOGaeyypa0JaeGymaeJaeGimaadabaGaem4AaS2aaSbaaSqaaiabiMda5aqabaGccqGH9aqpcqaIXaqmcqaIWaamaeaacqWGRbWAdaWgaaWcbaGaeGymaeJaeGimaadabeaakiabg2da9iabigdaXiabicdaWaaaaaa@624E@

and the dynamic response of the genetic regulatory network is shown in Figure 3(a). *X*_*e *_= [0.7339 0.7339 1 0.9283 0.9283]^*T *^is a stable equilibrium point of the genetic regulatory network. Since we are interested in the robust stability of the equilibrium *X*_*e *_= [0.7339 0.7339 1 0.9283 0.9283]^*T *^under stochastic parameter perturbation, the origin should be shifted to the equilibrium *X*_*e *_= [0.7339 0.7339 1 0.9283 0.9283]^*T *^. Then the genetic regulatory network should be rewritten under coordinate shift (see equation (A17)) and the dynamic response of the genetic regulatory network under coordinate shift is shown in Figure 3(b). It is seen that the origin is shifted to the equilibrium point *X*_*e*_, i.e., *x' *= 0 is a stable equilibrium point.

Suppose the genetic regulatory parameter suffer some stochastic perturbations shown as (A 18). In order to discuss the robust stability of the stochastic regulatory network in (A18) at the equilibrium point *X*_*e *_= [0.7339 0.7339 1 0.9283 0.9283]^*T*^, we can rewrite the genetic regulatory network under such perturbations by coordinate shift as (6) (shown in (A19)). Following remark 2(ii), we can globally linearize the system and solve the LMIs in (10) to see if the perturbed stochastic system is stable or not under this kind of stochastic intrinsic noise. In the perturbative case, we can find a positive definite *P *of the LMIs (shown in Appendix 5), so the stochastic system (A19) is stable in probability at *x' = 0 *under this intrinsic noise. The dynamic responses of the perturbed stochastic systems in (A18) and (A19) are shown in Figure 3(c) and Figure 3(d), respectively, which confirms the conclusion of our proposed computational method of robust stability.

Suppose the kinetic parameters suffer another stochastic perturbations shown in (A20). In order to discuss the robust stability of the stochastic regulatory network in (A20) at the equilibrium point *X*_*e *_= [0.7339 0.7339 1 0.9283 0.9283]^*T*^, we again rewrite the genetic regulatory network under such perturbations by coordinate shift as (A21). In this stochastic noise case, we can not find a positive definite *P *of the LMIs, so the stability of the stochastic regulatory system is not guaranteed. This result is consistent with the dynamic responses we present in Figures 3(e) and 3(f) for the stochastic system (A20) and the shifted stochastic system in (A21), respectively.

After validating the network robust stability under intrinsic fluctuations, we want to confirm the theorem in (21) about the estimation of the attenuation or amplification level *ρ*_*o *_of the extrinsic noise. Suppose the genetic regulatory network in (22) suffers an intrinsic stochastic kinetic parameter perturbation as (A18) and an extrinsic noise (shown in (A22)). In this case, *H *in (10) is an identity matrix. Let *υ*(*t*) = [*υ*_1_(*t*) *υ*_2_(*t*) *υ*_3_(*t*) *υ*_4_(*t*) *υ*_5_(*t*)]^*T *^denote the extrinsic noise vector. We are interested in the effects of extrinsic noise *υ*(*t*) on individual gene *i*, so we let *C *in (11) be [1 0 0 0 0], [0 1 0 0 0], [0 0 1 0 0], [0 0 0 1 0], [0 0 0 0 1], for gene 1, gene 2, gene 3, gene 4, gene 5, respectively. For the convenience of validation, we let *υ*(*t*) be white Gaussian noise with mean = 2 and variance = 1. By Remark 4(ii), we again globally linearize the system and solve the LMIs in (21) to calculate the attenuation (amplification) level *ρ*_*o*_. After solving the constrained optimization problem in (21), the optimal attenuation level *ρ*_*o *_of genes 1, 2, 3, 4, 5 are 0.5723, 0.5328, 0.5489, 0.6980, 0.6964, for *X*_1_, *X*_2_, *X*_3_, *X*_4 _and *X*_5_, respectively. This means that the noise attenuation levels of these genes can not exceed these values, respectively. Obviously, extrinsic noises are all attenuated at these genes in the gene network, so these genes are robust to these noises.

The dynamic response of the noise-driven case is shown in Figure 3(g). We can compute the L_2_-norm of *Z*(*t*) in Figure 3(g) to verify the attenuation level *ρ*_*o *_that we obtain by the computation method above. Thus, by the system simulation results, we get attenuatoion level ‖X1‖2‖υ‖2
 MathType@MTEF@5@5@+=feaafiart1ev1aaatCvAUfKttLearuWrP9MDH5MBPbIqV92AaeXatLxBI9gBaebbnrfifHhDYfgasaacH8akY=wiFfYdH8Gipec8Eeeu0xXdbba9frFj0=OqFfea0dXdd9vqai=hGuQ8kuc9pgc9s8qqaq=dirpe0xb9q8qiLsFr0=vr0=vr0dc8meaabaqaciaacaGaaeqabaqabeGadaaakeaadaWcaaqaamaafmaabaGaemiwaG1aaSbaaSqaaiabigdaXaqabaaakiaawMa7caGLkWoadaWgaaWcbaGaeGOmaidabeaaaOqaamaafmaabaacciGae8xXduhacaGLjWUaayPcSdWaaSbaaSqaaiabikdaYaqabaaaaaaa@397D@ ≈ 0.4954 < 0.5723 for gene 1, ‖X2‖2‖υ‖2
 MathType@MTEF@5@5@+=feaafiart1ev1aaatCvAUfKttLearuWrP9MDH5MBPbIqV92AaeXatLxBI9gBaebbnrfifHhDYfgasaacH8akY=wiFfYdH8Gipec8Eeeu0xXdbba9frFj0=OqFfea0dXdd9vqai=hGuQ8kuc9pgc9s8qqaq=dirpe0xb9q8qiLsFr0=vr0=vr0dc8meaabaqaciaacaGaaeqabaqabeGadaaakeaadaWcaaqaamaafmaabaGaemiwaG1aaSbaaSqaaiabikdaYaqabaaakiaawMa7caGLkWoadaWgaaWcbaGaeGOmaidabeaaaOqaamaafmaabaacciGae8xXduhacaGLjWUaayPcSdWaaSbaaSqaaiabikdaYaqabaaaaaaa@397F@ ≈ 0.5133 < 0.5328 for gene 2, ‖X3‖2‖υ‖2
 MathType@MTEF@5@5@+=feaafiart1ev1aaatCvAUfKttLearuWrP9MDH5MBPbIqV92AaeXatLxBI9gBaebbnrfifHhDYfgasaacH8akY=wiFfYdH8Gipec8Eeeu0xXdbba9frFj0=OqFfea0dXdd9vqai=hGuQ8kuc9pgc9s8qqaq=dirpe0xb9q8qiLsFr0=vr0=vr0dc8meaabaqaciaacaGaaeqabaqabeGadaaakeaadaWcaaqaamaafmaabaGaemiwaG1aaSbaaSqaaiabiodaZaqabaaakiaawMa7caGLkWoadaWgaaWcbaGaeGOmaidabeaaaOqaamaafmaabaacciGae8xXduhacaGLjWUaayPcSdWaaSbaaSqaaiabikdaYaqabaaaaaaa@3981@ ≈ 0.4916 < 0.5489 for gene 3, ‖X4‖2‖υ‖2
 MathType@MTEF@5@5@+=feaafiart1ev1aaatCvAUfKttLearuWrP9MDH5MBPbIqV92AaeXatLxBI9gBaebbnrfifHhDYfgasaacH8akY=wiFfYdH8Gipec8Eeeu0xXdbba9frFj0=OqFfea0dXdd9vqai=hGuQ8kuc9pgc9s8qqaq=dirpe0xb9q8qiLsFr0=vr0=vr0dc8meaabaqaciaacaGaaeqabaqabeGadaaakeaadaWcaaqaamaafmaabaGaemiwaG1aaSbaaSqaaiabisda0aqabaaakiaawMa7caGLkWoadaWgaaWcbaGaeGOmaidabeaaaOqaamaafmaabaacciGae8xXduhacaGLjWUaayPcSdWaaSbaaSqaaiabikdaYaqabaaaaaaa@3983@ ≈ 0.6281 < 0.6980 for gene 4, and ‖X5‖2‖υ‖2
 MathType@MTEF@5@5@+=feaafiart1ev1aaatCvAUfKttLearuWrP9MDH5MBPbIqV92AaeXatLxBI9gBaebbnrfifHhDYfgasaacH8akY=wiFfYdH8Gipec8Eeeu0xXdbba9frFj0=OqFfea0dXdd9vqai=hGuQ8kuc9pgc9s8qqaq=dirpe0xb9q8qiLsFr0=vr0=vr0dc8meaabaqaciaacaGaaeqabaqabeGadaaakeaadaWcaaqaamaafmaabaGaemiwaG1aaSbaaSqaaiabiwda1aqabaaakiaawMa7caGLkWoadaWgaaWcbaGaeGOmaidabeaaaOqaamaafmaabaacciGae8xXduhacaGLjWUaayPcSdWaaSbaaSqaaiabikdaYaqabaaaaaaa@3985@ ≈ 0.6424 < 0.6954 for gene 5, respectively. Obviously, the inequality in (14) holds, i.e., the proposed optimal noise attenuation levels are the upper bounds of those computed by the simulation results. In this genetic regulatory network, we found that the noises at *X*_1_*, X*_2_*, X*_3_*, X*_4_, and *X*_5 _are all attenuated by the network, which are validated by both the proposed optimal attenuation measure method and system simulation.

In the stochastic genetic regulatory system (A22), *υ*(*t*) can not only be of extrinsic noise but also be of extrinsic signal such as sinusoid signal. In this situation, we let *υ*(*t*) in (A22) be 2+sin(10t) to verify the attenuation levels of extrinsic signal from the environment. The dynamic response of the signal-driven case is shown in Figure 3(h). By the simulation results, we can also obtain the attenuation level ‖X1‖2‖υ‖2
 MathType@MTEF@5@5@+=feaafiart1ev1aaatCvAUfKttLearuWrP9MDH5MBPbIqV92AaeXatLxBI9gBaebbnrfifHhDYfgasaacH8akY=wiFfYdH8Gipec8Eeeu0xXdbba9frFj0=OqFfea0dXdd9vqai=hGuQ8kuc9pgc9s8qqaq=dirpe0xb9q8qiLsFr0=vr0=vr0dc8meaabaqaciaacaGaaeqabaqabeGadaaakeaadaWcaaqaamaafmaabaGaemiwaG1aaSbaaSqaaiabigdaXaqabaaakiaawMa7caGLkWoadaWgaaWcbaGaeGOmaidabeaaaOqaamaafmaabaacciGae8xXduhacaGLjWUaayPcSdWaaSbaaSqaaiabikdaYaqabaaaaaaa@397D@ ≈ 0.4895 < 0.5723 for gene 1, ‖X2‖2‖υ‖2
 MathType@MTEF@5@5@+=feaafiart1ev1aaatCvAUfKttLearuWrP9MDH5MBPbIqV92AaeXatLxBI9gBaebbnrfifHhDYfgasaacH8akY=wiFfYdH8Gipec8Eeeu0xXdbba9frFj0=OqFfea0dXdd9vqai=hGuQ8kuc9pgc9s8qqaq=dirpe0xb9q8qiLsFr0=vr0=vr0dc8meaabaqaciaacaGaaeqabaqabeGadaaakeaadaWcaaqaamaafmaabaGaemiwaG1aaSbaaSqaaiabikdaYaqabaaakiaawMa7caGLkWoadaWgaaWcbaGaeGOmaidabeaaaOqaamaafmaabaacciGae8xXduhacaGLjWUaayPcSdWaaSbaaSqaaiabikdaYaqabaaaaaaa@397F@ ≈ 0.5113 < 0.5328 for gene 2, ‖X3‖2‖υ‖2
 MathType@MTEF@5@5@+=feaafiart1ev1aaatCvAUfKttLearuWrP9MDH5MBPbIqV92AaeXatLxBI9gBaebbnrfifHhDYfgasaacH8akY=wiFfYdH8Gipec8Eeeu0xXdbba9frFj0=OqFfea0dXdd9vqai=hGuQ8kuc9pgc9s8qqaq=dirpe0xb9q8qiLsFr0=vr0=vr0dc8meaabaqaciaacaGaaeqabaqabeGadaaakeaadaWcaaqaamaafmaabaGaemiwaG1aaSbaaSqaaiabiodaZaqabaaakiaawMa7caGLkWoadaWgaaWcbaGaeGOmaidabeaaaOqaamaafmaabaacciGae8xXduhacaGLjWUaayPcSdWaaSbaaSqaaiabikdaYaqabaaaaaaa@3981@ ≈ 0.4831 < 0.5489 for gene 3, ‖X4‖2‖υ‖2
 MathType@MTEF@5@5@+=feaafiart1ev1aaatCvAUfKttLearuWrP9MDH5MBPbIqV92AaeXatLxBI9gBaebbnrfifHhDYfgasaacH8akY=wiFfYdH8Gipec8Eeeu0xXdbba9frFj0=OqFfea0dXdd9vqai=hGuQ8kuc9pgc9s8qqaq=dirpe0xb9q8qiLsFr0=vr0=vr0dc8meaabaqaciaacaGaaeqabaqabeGadaaakeaadaWcaaqaamaafmaabaGaemiwaG1aaSbaaSqaaiabisda0aqabaaakiaawMa7caGLkWoadaWgaaWcbaGaeGOmaidabeaaaOqaamaafmaabaacciGae8xXduhacaGLjWUaayPcSdWaaSbaaSqaaiabikdaYaqabaaaaaaa@3983@ ≈ 0.6080 < 0.6980 for gene 4, and ‖X5‖2‖υ‖2
 MathType@MTEF@5@5@+=feaafiart1ev1aaatCvAUfKttLearuWrP9MDH5MBPbIqV92AaeXatLxBI9gBaebbnrfifHhDYfgasaacH8akY=wiFfYdH8Gipec8Eeeu0xXdbba9frFj0=OqFfea0dXdd9vqai=hGuQ8kuc9pgc9s8qqaq=dirpe0xb9q8qiLsFr0=vr0=vr0dc8meaabaqaciaacaGaaeqabaqabeGadaaakeaadaWcaaqaamaafmaabaGaemiwaG1aaSbaaSqaaiabiwda1aqabaaakiaawMa7caGLkWoadaWgaaWcbaGaeGOmaidabeaaaOqaamaafmaabaacciGae8xXduhacaGLjWUaayPcSdWaaSbaaSqaaiabikdaYaqabaaaaaaa@3985@ ≈ 0.6227 < 0.6964 for gene 5, respectively, which are also consistent with the optimal attenuation levels calculated by the method we proposed.

## Discussion

Genetic and biochemical networks are influenced by unavoidable fluctuations which cause random perturbations of biochemical parameters. These perturbations are reflected in dynamic models as numerical changes in reaction rate constants of the system. Genetic regulatory networks also suffer from environmental disturbances. We classify these perturbations and disturbances as intrinsic and extrinsic noise which are modeled as state dependent noise and state independent noise, respectively. In this study, we propose a method to examine the network robust stability under intrinsic noise and to measure the attenuation or amplification of extrinsic noise. We found that the dynamic stability of a nonlinear perturbative genetic regulatory network under intrinsic noise is guaranteed if we can find a Lyapunov function which satisfies the Hamilton Jacobi inequality (HJI) in (8). We can also estimate the attenuation (amplification) level of the nonlinear perturbative genetic regulatory network if we can find a Lyapunov function which satisfies the HJI in (19). The robustness measurement of S-systems in biochemical network [33] is based on the deterministic parameter perturbations at steady state. The attenuation or amplification of extrinsic noises has not been considered. This study considers the robust stability problem of a more general gene regulatory network with intrinsic and extrinsic noises.

The effect of different types of noise on biochemical networks or genetic regulatory networks is studied based on the robust stability and noise attenuation (amplification) from the stochastic system perspective. The proposed method can work on both linear and nonlinear biosystems. In addition, it can be applied to all kinds of model types. Once we model the genetic regulatory or biochemical network, we can analyze the robust stability and noise attenuation by the methods proposed here. In general, it is not easy to solve HJI in (8) to find the robust stability of genetic regulatory networks under intrinsic noise, or to solve HJI in (19) to get the attenuation level or amplification level to extrinsic noise. In order to avoid solving the HJI in a nonlinear stochastic gene regulatory network, which is not easy to solve directly, we replace the HJI by global linearization with a set of linear matrix inequalities (LMIs). We can easily solve the LMIs by Matlab LMI Toolbox. Therefore, we can analyze the effect of noises on each gene of a genetic regulatory network more efficiently.

This method provides a way to determine whether the robust stability of the genetic regulatory network is guaranteed under intrinsic fluctuations. Furthermore, we could also estimate to what extent the fluctuations can cease the stability of the network. Therefore, we can gain more insight into how the genetic regulatory network is robustly stabilized. It also provides a quantitative measurement to see to what extent the extrinsic noise is attenuated or amplified at each gene in the network. By choosing different values for *C *in (11) or (12) from [1 0 0…0 0] to [0 0 0…0…0 1], we can quantify the attenuation or amplification level of extrinsic noises at different genes in a genetic regulatory network. We can also compare the attenuation or amplification levels of noises at different networks by choosing *C *as *I*, the identity matrix.

From LMI in (7) or (10), in order to let *P > *0, it is seen that there are two schemes for genetic regulatory networks to improve the robust stability against intrinsic noise. One scheme is to make the eigenvalues of the system matrix *N *as negative as possible (i.e., the eigenvalues (or system poles) *of N *are far to the left hand side of the complex domain) so that large parameter variation matrix *M *could be tolerated [9]. Adequate negative feedbacks in networks could place the eigenvalues of *N *to far left hand side of the complex domain to achieve this purpose. Another scheme is to make the parameter variation matrix *M *as small as possible so that the robust stability condition (7) or (10) is not violated. Pervasive redundancy in genetic regulatory network could achieve this purpose [9].

Similarly, from (16) or (17), in order to let *P > *0, we examine the condition where *N *is more negative, and *M *and *H *are smaller, i.e., the eigenvalues of system matrix *N *are all far to the left hand side in the complex domain, the magnitude of intrinsic noise is smaller, and the coupling between the network and environment is weak. In this case, the genetic regulatory network will attenuate the extrinsic noise to a smaller level.

Our noise analysis has been confirmed by the typical genetic regulatory network with numerical simulations. From the simulations, we found that if we can find a common *P > *0 of LMIs in (10), then the robust stability of the network is guaranteed. Therefore, the network can tolerate this kind of noises. Moreover, we also found that we can calculate the attenuation or amplification level of noises for each gene by solving LMIs in (18) or (21). Comparing the results of attenuation (or amplification) level by the proposed analysis method with those by the true system simulations, however, we find that there are still some discrepancies. This is because the HJI problem is replaced by solving LMIs, which are only an approximation method and may lead to a conservative result and the proposed optimal attenuation measurements are the upper bounds of those computed by the simulation results.

Once we could analyze the effects of the noises on the genetic regulatory network, we could know which genes are influenced much by noise, i.e., are more sensitive to noise. These genes are at the locations which we call the weak structure of the network. This information could provide potential therapeutic targets for treatment of disease. Understanding the effects of noise and the weakness of the network is helpful for improving the stability robustness or achieving a desired noise attenuation or amplification of the genetic regulatory network, on which a drug design could be based. In the future, we may be able to design drugs or construct some other pathways by transfection and transformation biotechnologies to improve the robustness of weak structures of gene networks, so that genetic regulatory networks or biochemical networks can be protected from the influence of the environmental extrinsic noise and the perturbative intrinsic noise.

## Conclusion

In this study, a nonlinear stochastic system is developed to model a gene regulatory network under intrinsic noise and extrinsic noise. Based on the stochastic stability, the robust stability condition is developed as in Theorem 1 for linear stochastic gene regulatory network and in Theorem 2 for nonlinear gene regulatory network. By the global linearization method, the robust stability condition of nonlinear gene regulatory network is reduced by solving LMIs in (10) for the convenience of computation.

Based on the robust H_∞ _filtering theory, the amplification or attenuation of extrinsic noise at an interested gene is to solve the minimum *ρ*_*o *_in (18) for linear stochastic gene regulatory network and *ρ*_*o *_in (2) for nonlinear stochastic gene regulatory network, which could be reduced to the constrained optimization problem in (21). If *ρ*_*o *_< 1, the extrinsic noise is attenuated at that gene and if *ρ*_*o *_> 1, the extrinsic noise is amplified at that gene. The genes with *ρ*_*o *_< 1 are more robust than the genes with *ρ*_*o *_> 1. If *ρ*_*o *_of a gene is much larger than 1, it should be significantly affected by extrinsic noises and is at a weak structure of the gene network. The robust filtering analysis will be potential for robust gene circuit design in future, on which gene therapy and drug design could be based.

The future work will focus on gene circuit control design method to improve the robust stability of nonlinear gene network to tolerate the much large intrinsic noise and to achieve a prescribed attenuation level *ρ*_*o *_for a desired extrinsic noise filtering. These robust circuit design methods are useful for the biotechnological purpose or the therapeutic purpose.

## Methods

The stochastic stability of the Langevin equation (4) for nonlinear stochastic regulatory networks is discussed by the stochastic Lyapunov theory [35,39]. Suppose *V*(*x*) > 0 with *V*(0) = 0 denotes the Lyapunov function of the Langevin equation in (4). Then the equilibrium point *x *= 0 of the stochastic gene regulatory network is stable in probability under intrinsic noise if the total power could not be increase, i.e., the inequality (5) should hold. In the linear stochastic gene regulatory network in (3), the robust stability is reduced to the existence of *P *> 0 in the LMI of (7) in Theorem 1. By the global linearization, the robust stability condition for nonlinear stochastic gene regulatory network is reduced to the existence of a common *P > *0 in the LMIs in (10), which could be easily solved by the LMI toolbox in Matlab.

The stochastic filtering of extrinsic noise *υ*(*t*) in the nonlinear stochastic gene network is considered from the nonlinear H_∞ _filtering theory of nonlinear stochastic system [15], i.e., the effect of extrinsic noise on an interested gene *Z*(*t*) should be equal to or less than a level *ρ*_*o*_. That means the inequality (14) or (15) should hold. Then the filtering problem for the nonlinear stochastic gene regulatory network under intrinsic noise and extrinsic noise is to solve the constrained optimization problem in (20) to obtain *ρ*_*o*_. If *ρ*_*o *_< 1, then the extrinsic noise is attenuated and if *ρ*_*o *_> 1, then the extrinsic noise is amplified. In the linear stochastic gene regulatory network in (11), the filtering problem is reduced to solving the constrained optimization problem in (18). By the global linearization method, the nonlinear filtering problem in (20) is reduced to the constrained optimization problem in (21), which could be easily solved by LMI toolbox in Matlab.

## Appendix

### Appendix 1: proof of Theorem 1

Let us denote the Lyapunov (energy) function of linear stochastic regulatory network in equation (2) as *V*(*x*) = *x*(*t*)^*T*^*Px*(*t*), for some symmetric positive definite matrix *P*^*T *^= *P *> 0. Then the change of Lyapunov function is obtained by following Itô derivative [36,39]

E{ddtV(x)}=E{(∂∂xV(x))TNx(t)+12xT(t)MT∂2V(x)∂x2Mx(t)}=E{xT(t)PNx(t)+xT(t)NTPx(t)+xT(t)MTPMx(t)}=E{xT(t)(PN+NTP+MTPM)x(t)}     (A1)
 MathType@MTEF@5@5@+=feaafiart1ev1aaatCvAUfKttLearuWrP9MDH5MBPbIqV92AaeXatLxBI9gBaebbnrfifHhDYfgasaacH8akY=wiFfYdH8Gipec8Eeeu0xXdbba9frFj0=OqFfea0dXdd9vqai=hGuQ8kuc9pgc9s8qqaq=dirpe0xb9q8qiLsFr0=vr0=vr0dc8meaabaqaciaacaGaaeqabaqabeGadaaakeaafaqadeWabaaabaGaemyrau0aaiWaaeaadaWcaaqaaiabdsgaKbqaaiabdsgaKjabdsha0baacqWGwbGvcqGGOaakcqWG4baEcqGGPaqkaiaawUhacaGL9baacqGH9aqpcqWGfbqrdaGadaqaamaabmaabaWaaSaaaeaaiiGacqWFciITaeaacqWFciITcqWG4baEaaGaemOvayLaeiikaGIaemiEaGNaeiykaKcacaGLOaGaayzkaaWaaWbaaSqabeaacqWGubavaaGccqWGobGtcqWG4baEcqGGOaakcqWG0baDcqGGPaqkcqGHRaWkdaWcaaqaaiabigdaXaqaaiabikdaYaaacqWG4baEdaahaaWcbeqaaiabdsfaubaakiabcIcaOiabdsha0jabcMcaPiabd2eannaaCaaaleqabaGaemivaqfaaOWaaSaaaeaacqWFciITdaahaaWcbeqaaiabikdaYaaakiabdAfawjabcIcaOiabdIha4jabcMcaPaqaaiab=jGi2kabdIha4naaCaaaleqabaGaeGOmaidaaaaakiabd2eanjabdIha4jabcIcaOiabdsha0jabcMcaPaGaay5Eaiaaw2haaaqaaiabg2da9iabdweafnaacmaabaGaemiEaG3aaWbaaSqabeaacqWGubavaaGccqGGOaakcqWG0baDcqGGPaqkcqWGqbaucqWGobGtcqWG4baEcqGGOaakcqWG0baDcqGGPaqkcqGHRaWkcqWG4baEdaahaaWcbeqaaiabdsfaubaakiabcIcaOiabdsha0jabcMcaPiabd6eaonaaCaaaleqabaGaemivaqfaaOGaemiuaaLaemiEaGNaeiikaGIaemiDaqNaeiykaKIaey4kaSIaemiEaG3aaWbaaSqabeaacqWGubavaaGccqGGOaakcqWG0baDcqGGPaqkcqWGnbqtdaahaaWcbeqaaiabdsfaubaakiabdcfaqjabd2eanjabdIha4jabcIcaOiabdsha0jabcMcaPaGaay5Eaiaaw2haaaqaaiabg2da9iabdweafnaacmaabaGaemiEaG3aaWbaaSqabeaacqWGubavaaGccqGGOaakcqWG0baDcqGGPaqkdaqadaqaaiabdcfaqjabd6eaojabgUcaRiabd6eaonaaCaaaleqabaGaemivaqfaaOGaemiuaaLaey4kaSIaemyta00aaWbaaSqabeaacqWGubavaaGccqWGqbaucqWGnbqtaiaawIcacaGLPaaacqWG4baEcqGGOaakcqWG0baDcqGGPaqkaiaawUhacaGL9baaaaGaaCzcaiaaxMaadaqadaqaaGqaaiab+feabjabigdaXaGaayjkaiaawMcaaaaa@BCF5@

From the inequality (7), we get

E{ddtV(x)}≤0     (A2)
 MathType@MTEF@5@5@+=feaafiart1ev1aaatCvAUfKttLearuWrP9MDH5MBPbIqV92AaeXatLxBI9gBaebbnrfifHhDYfgasaacH8akY=wiFfYdH8Gipec8Eeeu0xXdbba9frFj0=OqFfea0dXdd9vqai=hGuQ8kuc9pgc9s8qqaq=dirpe0xb9q8qiLsFr0=vr0=vr0dc8meaabaqaciaacaGaaeqabaqabeGadaaakeaacqWGfbqrdaGadaqaamaalaaabaGaemizaqgabaGaemizaqMaemiDaqhaaiabdAfawjabcIcaOiabdIha4jabcMcaPaGaay5Eaiaaw2haaiabgsMiJkabicdaWiaaxMaacaWLjaWaaeWaaeaaieaacqWFbbqqcqaIYaGmaiaawIcacaGLPaaaaaa@3FE5@

Since the change of Lyapunov (energy) function is nonpositive, the linear stochastic genetic regulatory network is stable in probability.

### Appendix 2: proof of Theorem 2

Let us denote the Lyapunov function *V(x) *of the perturbative nonlinear genetic regulatory network. By following Itô derivative, we get [15,37]

E{ddtV(x)}=E{(∂∂xV(x))TN(x(t))+12MT(x(t))∂2V(x)∂x2M(x(t))}     (A3)
 MathType@MTEF@5@5@+=feaafiart1ev1aaatCvAUfKttLearuWrP9MDH5MBPbIqV92AaeXatLxBI9gBaebbnrfifHhDYfgasaacH8akY=wiFfYdH8Gipec8Eeeu0xXdbba9frFj0=OqFfea0dXdd9vqai=hGuQ8kuc9pgc9s8qqaq=dirpe0xb9q8qiLsFr0=vr0=vr0dc8meaabaqaciaacaGaaeqabaqabeGadaaakeaacqWGfbqrdaGadaqaamaalaaabaGaemizaqgabaGaemizaqMaemiDaqhaaiabdAfawjabcIcaOiabdIha4jabcMcaPaGaay5Eaiaaw2haaiabg2da9iabdweafnaacmaabaWaaeWaaeaadaWcaaqaaGGaciab=jGi2cqaaiab=jGi2kabdIha4baacqWGwbGvcqGGOaakcqWG4baEcqGGPaqkaiaawIcacaGLPaaadaahaaWcbeqaaiabdsfaubaakiabd6eaojabcIcaOiabdIha4jabcIcaOiabdsha0jabcMcaPiabcMcaPiabgUcaRmaalaaabaGaeGymaedabaGaeGOmaidaaiabd2eannaaCaaaleqabaGaemivaqfaaOGaeiikaGIaemiEaGNaeiikaGIaemiDaqNaeiykaKIaeiykaKYaaSaaaeaacqWFciITdaahaaWcbeqaaiabikdaYaaakiabdAfawjabcIcaOiabdIha4jabcMcaPaqaaiab=jGi2kabdIha4naaCaaaleqabaGaeGOmaidaaaaakiabd2eanjabcIcaOiabdIha4jabcIcaOiabdsha0jabcMcaPiabcMcaPaGaay5Eaiaaw2haaiaaxMaacaWLjaWaaeWaaeaaieaacqGFbbqqcqaIZaWmaiaawIcacaGLPaaaaaa@72BB@

By the inequality (8), we get

E{ddtV(x)}≤0     (A4)
 MathType@MTEF@5@5@+=feaafiart1ev1aaatCvAUfKttLearuWrP9MDH5MBPbIqV92AaeXatLxBI9gBaebbnrfifHhDYfgasaacH8akY=wiFfYdH8Gipec8Eeeu0xXdbba9frFj0=OqFfea0dXdd9vqai=hGuQ8kuc9pgc9s8qqaq=dirpe0xb9q8qiLsFr0=vr0=vr0dc8meaabaqaciaacaGaaeqabaqabeGadaaakeaacqWGfbqrdaGadaqaamaalaaabaGaemizaqgabaGaemizaqMaemiDaqhaaiabdAfawjabcIcaOiabdIha4jabcMcaPaGaay5Eaiaaw2haaiabgsMiJkabicdaWiaaxMaacaWLjaWaaeWaaeaacqqGbbqqcqaI0aanaiaawIcacaGLPaaaaaa@3FE2@

Then the nonlinear perturbative stochastic regulatory network in equation (4) is stable in probability.

### Appendix 3: proof of Theorem 3

Consider the following equality,

∫0∞ZT(t)Z(t)dt=V(x(0))−V(x(∞))+∫0∞(ZT(t)Z(t)+dV(x)dt)dt     (A5)
 MathType@MTEF@5@5@+=feaafiart1ev1aaatCvAUfKttLearuWrP9MDH5MBPbIqV92AaeXatLxBI9gBaebbnrfifHhDYfgasaacH8akY=wiFfYdH8Gipec8Eeeu0xXdbba9frFj0=OqFfea0dXdd9vqai=hGuQ8kuc9pgc9s8qqaq=dirpe0xb9q8qiLsFr0=vr0=vr0dc8meaabaqaciaacaGaaeqabaqabeGadaaakeaadaWdXaqaaiabdQfaAnaaCaaaleqabaGaemivaqfaaaqaaiabicdaWaqaaiabg6HiLcqdcqGHRiI8aOGaeiikaGIaemiDaqNaeiykaKIaemOwaOLaeiikaGIaemiDaqNaeiykaKIaemizaqMaemiDaqNaeyypa0JaemOvayLaeiikaGIaemiEaGNaeiikaGIaeGimaaJaeiykaKIaeiykaKIaeyOeI0IaemOvayLaeiikaGIaemiEaGNaeiikaGIaeyOhIuQaeiykaKIaeiykaKIaey4kaSYaa8qmaeaadaqadaqaaiabdQfaAnaaCaaaleqabaGaemivaqfaaOGaeiikaGIaemiDaqNaeiykaKIaemOwaOLaeiikaGIaemiDaqNaeiykaKIaey4kaSYaaSaaaeaacqWGKbazcqWGwbGvcqGGOaakcqWG4baEcqGGPaqkaeaacqWGKbazcqWG0baDaaaacaGLOaGaayzkaaGaemizaqMaemiDaqNaaCzcaiaaxMaadaqadaqaaGqaaiab=feabjabiwda1aGaayjkaiaawMcaaaWcbaGaeGimaadabaGaeyOhIukaniabgUIiYdaaaa@70A0@

By Itô formula for stochastic equation (11), we get

E{dV(x)dt}=E{(∂∂xV(x))T(Nx(t)+Hυ(t))+12xT(t)MT∂2V(x)∂x2Mx(t)}     (A6)
 MathType@MTEF@5@5@+=feaafiart1ev1aaatCvAUfKttLearuWrP9MDH5MBPbIqV92AaeXatLxBI9gBaebbnrfifHhDYfgasaacH8akY=wiFfYdH8Gipec8Eeeu0xXdbba9frFj0=OqFfea0dXdd9vqai=hGuQ8kuc9pgc9s8qqaq=dirpe0xb9q8qiLsFr0=vr0=vr0dc8meaabaqaciaacaGaaeqabaqabeGadaaakeaacqWGfbqrdaGadaqaamaalaaabaGaemizaqMaemOvayLaeiikaGIaemiEaGNaeiykaKcabaGaemizaqMaemiDaqhaaaGaay5Eaiaaw2haaiabg2da9iabdweafnaacmaabaWaaeWaaeaadaWcaaqaaGGaciab=jGi2cqaaiab=jGi2kabdIha4baacqWGwbGvcqGGOaakcqWG4baEcqGGPaqkaiaawIcacaGLPaaadaahaaWcbeqaaiabdsfaubaakmaabmaabaGaemOta4KaemiEaGNaeiikaGIaemiDaqNaeiykaKIaey4kaSIaemisaGKae8xXduNaeiikaGIaemiDaqNaeiykaKcacaGLOaGaayzkaaGaey4kaSYaaSaaaeaacqaIXaqmaeaacqaIYaGmaaGaemiEaG3aaWbaaSqabeaacqWGubavaaGccqGGOaakcqWG0baDcqGGPaqkcqWGnbqtdaahaaWcbeqaaiabdsfaubaakmaalaaabaGae8NaIy7aaWbaaSqabeaacqWFYaGmaaGccqWGwbGvcqGGOaakcqWG4baEcqGGPaqkaeaacqWFciITcqWG4baEdaahaaWcbeqaaiabikdaYaaaaaGccqWGnbqtcqWG4baEcqGGOaakcqWG0baDcqGGPaqkaiaawUhacaGL9baacaWLjaGaaCzcamaabmaabaacbaGae4xqaeKaeGOnaydacaGLOaGaayzkaaaaaa@7773@

Let us choose the Lyapunov function as *V*(*x*) = *x*(*t*)^*T *^*Px*(*t*). Then

E{dV(x)dt}=E{2xT(t)P(Nx(t)+Hυ(t))+xT(t)MTPMx(t)}=Ε{xT(t)P(Nx(t)+Hυ(t))+(xT(t)NT+υT(t)HT)Px(t)+xT(t)MTPMx(t)}     (A7)
 MathType@MTEF@5@5@+=feaafiart1ev1aaatCvAUfKttLearuWrP9MDH5MBPbIqV92AaeXatLxBI9gBaebbnrfifHhDYfgasaacH8akY=wiFfYdH8Gipec8Eeeu0xXdbba9frFj0=OqFfea0dXdd9vqai=hGuQ8kuc9pgc9s8qqaq=dirpe0xb9q8qiLsFr0=vr0=vr0dc8meaabaqaciaacaGaaeqabaqabeGadaaakeaafaqadeGabaaabaGaemyrau0aaiWaaeaadaWcaaqaaiabdsgaKjabdAfawjabcIcaOiabdIha4jabcMcaPaqaaiabdsgaKjabdsha0baaaiaawUhacaGL9baacqGH9aqpcqWGfbqrdaGadaqaaiabikdaYiabdIha4naaCaaaleqabaGaemivaqfaaOGaeiikaGIaemiDaqNaeiykaKIaemiuaa1aaeWaaeaacqWGobGtcqWG4baEcqGGOaakcqWG0baDcqGGPaqkcqGHRaWkcqWGibasiiGacqWFfpqDcqGGOaakcqWG0baDcqGGPaqkaiaawIcacaGLPaaacqGHRaWkcqWG4baEdaahaaWcbeqaaiabdsfaubaakiabcIcaOiabdsha0jabcMcaPiabd2eannaaCaaaleqabaGaemivaqfaaOGaemiuaaLaemyta0KaemiEaGNaeiikaGIaemiDaqNaeiykaKcacaGL7bGaayzFaaaabaGae8xpa0Jae8xLdu0aaiWaaeaaieGacqGF4baEdaahaaWcbeqaaiab+rfaubaakiabcIcaOiabdsha0jabcMcaPiabdcfaqnaabmaabaGaemOta4KaemiEaGNaeiikaGIaemiDaqNaeiykaKIaey4kaSIaemisaGKae8xXduNaeiikaGIaemiDaqNaeiykaKcacaGLOaGaayzkaaGaey4kaSYaaeWaaeaacqWG4baEdaahaaWcbeqaaiabdsfaubaakiabcIcaOiabdsha0jabcMcaPiabd6eaonaaCaaaleqabaGaemivaqfaaOGaey4kaSIae8xXdu3aaWbaaSqabeaacqWGubavaaGccqGGOaakcqWG0baDcqGGPaqkcqWGibasdaahaaWcbeqaaiabdsfaubaaaOGaayjkaiaawMcaaiabdcfaqjabdIha4jabcIcaOiabdsha0jabcMcaPiabgUcaRiabdIha4naaCaaaleqabaGaemivaqfaaOGaeiikaGIaemiDaqNaeiykaKIaemyta00aaWbaaSqabeaacqWGubavaaGccqWGqbaucqWGnbqtcqWG4baEcqGGOaakcqWG0baDcqGGPaqkaiaawUhacaGL9baaaaGaaCzcaiaaxMaadaqadaqaaGqaaiab9feabjabiEda3aGaayjkaiaawMcaaaaa@AD05@

Taking expectation to both sides of (A5) and substituting equation (A7) into equation (A5), we get

E{∫0∞ZT(t)Z(t)dt}=E{V(x(0))}−E{V(x(∞))}+E{∫0∞xT(t)CTCx(t)+xT(t)PNx(t)+xT(t)PHυ(t)+xT(t)NTPx(t)+υT(t)HTPx(t)+xT(t)MTPMx(t)dt}     (A8)
 MathType@MTEF@5@5@+=feaafiart1ev1aaatCvAUfKttLearuWrP9MDH5MBPbIqV92AaeXatLxBI9gBaebbnrfifHhDYfgasaacH8akY=wiFfYdH8Gipec8Eeeu0xXdbba9frFj0=OqFfea0dXdd9vqai=hGuQ8kuc9pgc9s8qqaq=dirpe0xb9q8qiLsFr0=vr0=vr0dc8meaabaqaciaacaGaaeqabaqabeGadaaakeaafaqadeGabaaabaGaemyrau0aaiWaaeaadaWdXaqaaiabdQfaAnaaCaaaleqabaGaemivaqfaaOGaeiikaGIaemiDaqNaeiykaKIaemOwaOLaeiikaGIaemiDaqNaeiykaKIaemizaqMaemiDaqhaleaacqaIWaamaeaacqGHEisPa0Gaey4kIipaaOGaay5Eaiaaw2haaiabg2da9iabdweafnaacmaabaGaemOvayLaeiikaGIaemiEaGNaeiikaGIaeGimaaJaeiykaKIaeiykaKcacaGL7bGaayzFaaGaeyOeI0Iaemyrau0aaiWaaeaacqWGwbGvcqGGOaakcqWG4baEcqGGOaakcqGHEisPcqGGPaqkcqGGPaqkaiaawUhacaGL9baacqGHRaWkcqWGfbqrdaGabaqaamaapedabaGaemiEaG3aaWbaaSqabeaacqWGubavaaaabaGaeGimaadabaGaeyOhIukaniabgUIiYdGccqGGOaakcqWG0baDcqGGPaqkcqWGdbWqdaahaaWcbeqaaiabdsfaubaakiabdoeadjabdIha4jabcIcaOiabdsha0jabcMcaPiabgUcaRiabdIha4naaCaaaleqabaGaemivaqfaaOGaeiikaGIaemiDaqNaeiykaKIaemiuaaLaemOta4KaemiEaGNaeiikaGIaemiDaqNaeiykaKcacaGL7baaaeaadaGacaqaaiabgUcaRiabdIha4naaCaaaleqabaGaemivaqfaaOGaeiikaGIaemiDaqNaeiykaKIaemiuaaLaemisaGecciGae8xXduNaeiikaGIaemiDaqNaeiykaKIaey4kaSIaemiEaG3aaWbaaSqabeaacqWGubavaaGccqGGOaakcqWG0baDcqGGPaqkcqWGobGtdaahaaWcbeqaaiabdsfaubaakiabdcfaqjabdIha4jabcIcaOiabdsha0jabcMcaPiabgUcaRiab=v8a1naaCaaaleqabaGaemivaqfaaOGaeiikaGIaemiDaqNaeiykaKIaemisaG0aaWbaaSqabeaacqWGubavaaGccqWGqbaucqWG4baEcqGGOaakcqWG0baDcqGGPaqkcqGHRaWkcqWG4baEdaahaaWcbeqaaiabdsfaubaakiabcIcaOiabdsha0jabcMcaPiabd2eannaaCaaaleqabaGaemivaqfaaOGaemiuaaLaemyta0KaemiEaGNaeiikaGIaemiDaqNaeiykaKIaemizaqMaemiDaqhacaGL9baaaaGaaCzcaiaaxMaadaqadaqaaGqaaiab+feabjabiIda4aGaayjkaiaawMcaaaaa@C1D3@

By the fact that

*X*^*T*^*Y *+ *Y*^*T*^*X *≤ *ρ*^2 ^*XX*^*T *^+ *ρ*^-2^*Y*^*T*^*Y*, for any *ρ*^2 ^> 0     (A9)

and *E*{*V*(*x*(∞))} ≥ 0, we get

E{∫0∞ZT(t)Z(t)dt}≤E{V(x(0))}+E{∫0∞xT(t)(PN+NTP+CTC+1ρ2PHHTP+MTPM)x(t)+ρ2υT(t)υ(t)dt}     (A10)
 MathType@MTEF@5@5@+=feaafiart1ev1aaatCvAUfKttLearuWrP9MDH5MBPbIqV92AaeXatLxBI9gBaebbnrfifHhDYfgasaacH8akY=wiFfYdH8Gipec8Eeeu0xXdbba9frFj0=OqFfea0dXdd9vqai=hGuQ8kuc9pgc9s8qqaq=dirpe0xb9q8qiLsFr0=vr0=vr0dc8meaabaqaciaacaGaaeqabaqabeGadaaakeaafaqaceGabaaabaGaemyrau0aaiWaaeaadaWdXaqaaiabdQfaAnaaCaaaleqabaGaemivaqfaaaqaaiabicdaWaqaaiabg6HiLcqdcqGHRiI8aOGaeiikaGIaemiDaqNaeiykaKIaemOwaOLaeiikaGIaemiDaqNaeiykaKIaemizaqMaemiDaqhacaGL7bGaayzFaaGaeyizImQaemyrau0aaiWaaeaacqWGwbGvcqGGOaakcqWG4baEcqGGOaakcqaIWaamcqGGPaqkcqGGPaqkaiaawUhacaGL9baacqGHRaWkcqWGfbqrdaGabaqaamaapedabaGaemiEaG3aaWbaaSqabeaacqWGubavaaaabaGaeGimaadabaGaeyOhIukaniabgUIiYdGccqGGOaakcqWG0baDcqGGPaqkaiaawUhaamaabeaabaGaemiuaaLaemOta4Kaey4kaSIaemOta40aaWbaaSqabeaacqWGubavaaGccqWGqbaucqGHRaWkcqWGdbWqdaahaaWcbeqaaiabdsfaubaakiabdoeadjabgUcaRmaalaaabaGaeGymaedabaacciGae8xWdi3aaWbaaSqabeaacqaIYaGmaaaaaaGccaGLOaaacqWGqbaucqWGibascqWGibasdaahaaWcbeqaaiabdsfaubaakiabdcfaqbqaamaaciaabaWaaeGaaeaacqGHRaWkcqWGnbqtdaahaaWcbeqaaiabdsfaubaakiabdcfaqjabd2eanbGaayzkaaGaemiEaGNaeiikaGIaemiDaqNaeiykaKIaey4kaSIae8xWdi3aaWbaaSqabeaacqaIYaGmaaGccqWFfpqDdaahaaWcbeqaaiabdsfaubaakiabcIcaOiabdsha0jabcMcaPiab=v8a1jabcIcaOiabdsha0jabcMcaPiabdsgaKjabdsha0bGaayzFaaaaaiaaxMaacaWLjaWaaeWaaeaaieaacqGFbbqqcqaIXaqmcqaIWaamaiaawIcacaGLPaaaaaa@956E@

By the inequality (16), we get

E{∫0∞ZT(t)Z(t)dt}≤E{V(x(0))}+ρ2E{∫0∞υT(t)υ(t)dt}     (A11)
 MathType@MTEF@5@5@+=feaafiart1ev1aaatCvAUfKttLearuWrP9MDH5MBPbIqV92AaeXatLxBI9gBaebbnrfifHhDYfgasaacH8akY=wiFfYdH8Gipec8Eeeu0xXdbba9frFj0=OqFfea0dXdd9vqai=hGuQ8kuc9pgc9s8qqaq=dirpe0xb9q8qiLsFr0=vr0=vr0dc8meaabaqaciaacaGaaeqabaqabeGadaaakeaacqWGfbqrdaGadaqaamaapedabaGaemOwaO1aaWbaaSqabeaacqWGubavaaGccqGGOaakcqWG0baDcqGGPaqkcqWGAbGwcqGGOaakcqWG0baDcqGGPaqkcqWGKbazcqWG0baDaSqaaiabicdaWaqaaiabg6HiLcqdcqGHRiI8aaGccaGL7bGaayzFaaGaeyizImQaemyrau0aaiWaaeaacqWGwbGvcqGGOaakcqWG4baEcqGGOaakcqaIWaamcqGGPaqkcqGGPaqkaiaawUhacaGL9baacqGHRaWkiiGacqWFbpGCdaahaaWcbeqaaiabikdaYaaakiabdweafnaacmaabaWaa8qmaeaacqWFfpqDdaahaaWcbeqaaiabdsfaubaakiabcIcaOiabdsha0jabcMcaPiab=v8a1jabcIcaOiabdsha0jabcMcaPiabdsgaKjabdsha0bWcbaGaeGimaadabaGaeyOhIukaniabgUIiYdaakiaawUhacaGL9baacaWLjaGaaCzcaiabcIcaOGqaaiab+feabjabigdaXiabigdaXiabcMcaPaaa@6CE6@

or

‖Z(t)‖22≤E{V(x(0))}+ρ2‖υ(t)‖22     (A12)
 MathType@MTEF@5@5@+=feaafiart1ev1aaatCvAUfKttLearuWrP9MDH5MBPbIqV92AaeXatLxBI9gBaebbnrfifHhDYfgasaacH8akY=wiFfYdH8Gipec8Eeeu0xXdbba9frFj0=OqFfea0dXdd9vqai=hGuQ8kuc9pgc9s8qqaq=dirpe0xb9q8qiLsFr0=vr0=vr0dc8meaabaqaciaacaGaaeqabaqabeGadaaakeaadaqbdaqaaiabdQfaAjabcIcaOiabdsha0jabcMcaPaGaayzcSlaawQa7amaaDaaaleaacqaIYaGmaeaacqaIYaGmaaGccqGHKjYOcqWGfbqrdaGadaqaaiabdAfawjabcIcaOiabdIha4jabcIcaOiabicdaWiabcMcaPiabcMcaPaGaay5Eaiaaw2haaiabgUcaRGGaciab=f8aYnaaCaaaleqabaGaeGOmaidaaOWaauWaaeaacqWFfpqDcqGGOaakcqWG0baDcqGGPaqkaiaawMa7caGLkWoadaqhaaWcbaGaeGOmaidabaGaeGOmaidaaOGaaCzcaiaaxMaadaqadaqaaGqaaiab+feabjabigdaXiabikdaYaGaayjkaiaawMcaaaaa@55FE@

### Appendix 4: proof of Theorem 4

Consider the following equality,

∫0∞ZT(t)Z(t)dt=V(x(0))−V(x(∞))+∫0∞(ZT(t)Z(t)+dV(x)dt)dt     (A13)
 MathType@MTEF@5@5@+=feaafiart1ev1aaatCvAUfKttLearuWrP9MDH5MBPbIqV92AaeXatLxBI9gBaebbnrfifHhDYfgasaacH8akY=wiFfYdH8Gipec8Eeeu0xXdbba9frFj0=OqFfea0dXdd9vqai=hGuQ8kuc9pgc9s8qqaq=dirpe0xb9q8qiLsFr0=vr0=vr0dc8meaabaqaciaacaGaaeqabaqabeGadaaakeaadaWdXaqaaiabdQfaAnaaCaaaleqabaGaemivaqfaaOGaeiikaGIaemiDaqNaeiykaKIaemOwaOLaeiikaGIaemiDaqNaeiykaKIaemizaqMaemiDaqNaeyypa0JaemOvayLaeiikaGIaemiEaGNaeiikaGIaeGimaaJaeiykaKIaeiykaKIaeyOeI0IaemOvayLaeiikaGIaemiEaGNaeiikaGIaeyOhIuQaeiykaKIaeiykaKIaey4kaSYaa8qmaeaadaqadaqaaiabdQfaAnaaCaaaleqabaGaemivaqfaaOGaeiikaGIaemiDaqNaeiykaKIaemOwaOLaeiikaGIaemiDaqNaeiykaKIaey4kaSYaaSaaaeaacqWGKbazcqWGwbGvcqGGOaakcqWG4baEcqGGPaqkaeaacqWGKbazcqWG0baDaaaacaGLOaGaayzkaaGaemizaqMaemiDaqNaaCzcaiaaxMaacqGGOaakieaacqWFbbqqcqaIXaqmcqaIZaWmcqGGPaqkaSqaaiabicdaWaqaaiabg6HiLcqdcqGHRiI8aaWcbaGaeGimaadabaGaeyOhIukaniabgUIiYdaaaa@71C0@

By Itô formula for stochastic equation (12), we get

E{dv(x)dt}=E{(∂∂xV(x))T(N(x)+H(x)υ(t))+12MT(x)∂2V(x)∂x2M(x)}     (A14)
 MathType@MTEF@5@5@+=feaafiart1ev1aaatCvAUfKttLearuWrP9MDH5MBPbIqV92AaeXatLxBI9gBaebbnrfifHhDYfgasaacH8akY=wiFfYdH8Gipec8Eeeu0xXdbba9frFj0=OqFfea0dXdd9vqai=hGuQ8kuc9pgc9s8qqaq=dirpe0xb9q8qiLsFr0=vr0=vr0dc8meaabaqaciaacaGaaeqabaqabeGadaaakeaacqWGfbqrdaGadaqaamaalaaabaGaemizaqMaemODayNaeiikaGIaemiEaGNaeiykaKcabaGaemizaqMaemiDaqhaaaGaay5Eaiaaw2haaiabg2da9iabdweafnaacmaabaWaaeWaaeaadaWcaaqaaGGaciab=jGi2cqaaiab=jGi2kabdIha4baacqWGwbGvcqGGOaakcqWG4baEcqGGPaqkaiaawIcacaGLPaaadaahaaWcbeqaaiabdsfaubaakmaabmaabaGaemOta4KaeiikaGIaemiEaGNaeiykaKIaey4kaSIaemisaGKaeiikaGIaemiEaGNaeiykaKIae8xXduNaeiikaGIaemiDaqNaeiykaKcacaGLOaGaayzkaaGaey4kaSYaaSaaaeaacqaIXaqmaeaacqaIYaGmaaGaemyta00aaWbaaSqabeaacqWGubavaaGccqGGOaakcqWG4baEcqGGPaqkdaWcaaqaaiab=jGi2oaaCaaaleqabaGaeGOmaidaaOGaemOvayLaeiikaGIaemiEaGNaeiykaKcabaGae8NaIyRaemiEaG3aaWbaaSqabeaacqaIYaGmaaaaaOGaemyta0KaeiikaGIaemiEaGNaeiykaKcacaGL7bGaayzFaaGaaCzcaiaaxMaacqGGOaakieaacqGFbbqqcqaIXaqmcqaI0aancqGGPaqkaaa@7641@

Taking expectation to both sides of (A13) and substituting equation (A14) into equation (A13), we get

E{∫0∞ZT(t)Z(t)dt}=E{V(x(0))}−E{V(x(∞))}+E{∫0∞(xT(t)CTCx(t)+(∂∂xV(x))TN(x)+(∂∂xV(x))TH(x)υ(t)+12MT(x)∂2V(x)∂x2M(x))dt}     (A15)
 MathType@MTEF@5@5@+=feaafiart1ev1aaatCvAUfKttLearuWrP9MDH5MBPbIqV92AaeXatLxBI9gBaebbnrfifHhDYfgasaacH8akY=wiFfYdH8Gipec8Eeeu0xXdbba9frFj0=OqFfea0dXdd9vqai=hGuQ8kuc9pgc9s8qqaq=dirpe0xb9q8qiLsFr0=vr0=vr0dc8meaabaqaciaacaGaaeqabaqabeGadaaakeaafaqadeGabaaabaGaemyrau0aaiWaaeaadaWdXaqaaiabdQfaAnaaCaaaleqabaGaemivaqfaaOGaeiikaGIaemiDaqNaeiykaKIaemOwaOLaeiikaGIaemiDaqNaeiykaKIaemizaqMaemiDaqhaleaacqaIWaamaeaacqGHEisPa0Gaey4kIipaaOGaay5Eaiaaw2haaiabg2da9iabdweafnaacmaabaGaemOvayLaeiikaGIaemiEaGNaeiikaGIaeGimaaJaeiykaKIaeiykaKcacaGL7bGaayzFaaGaeyOeI0Iaemyrau0aaiWaaeaacqWGwbGvcqGGOaakcqWG4baEcqGGOaakcqGHEisPcqGGPaqkcqGGPaqkaiaawUhacaGL9baacqGHRaWkcqWGfbqrdaGabaqaamaapedabaWaaeqaaeaacqWG4baEdaahaaWcbeqaaiabdsfaubaakiabcIcaOiabdsha0jabcMcaPiabdoeadnaaCaaaleqabaGaemivaqfaaOGaem4qamKaemiEaGNaeiikaGIaemiDaqNaeiykaKcacaGLOaaaaSqaaiabicdaWaqaaiabg6HiLcqdcqGHRiI8aaGccaGL7baaaeaadaGacaqaamaabiaabaGaey4kaSYaaeWaaeaadaWcaaqaaGGaciab=jGi2cqaaiab=jGi2kabdIha4baacqWGwbGvcqGGOaakcqWG4baEcqGGPaqkaiaawIcacaGLPaaadaahaaWcbeqaaiabdsfaubaakiabd6eaojabcIcaOiabdIha4jabcMcaPiabgUcaRmaabmaabaWaaSaaaeaacqWFciITaeaacqWFciITcqWG4baEaaGaemOvayLaeiikaGIaemiEaGNaeiykaKcacaGLOaGaayzkaaWaaWbaaSqabeaacqWGubavaaGccqWGibascqGGOaakcqWG4baEcqGGPaqkcqWFfpqDcqGGOaakcqWG0baDcqGGPaqkcqGHRaWkdaWcaaqaaiabigdaXaqaaiabikdaYaaacqWGnbqtdaahaaWcbeqaaiabdsfaubaakiabcIcaOiabdIha4jabcMcaPmaalaaabaGae8NaIy7aaWbaaSqabeaacqaIYaGmaaGccqWGwbGvcqGGOaakcqWG4baEcqGGPaqkaeaacqWFciITcqWG4baEdaahaaWcbeqaaiabikdaYaaaaaGccqWGnbqtcqGGOaakcqWG4baEcqGGPaqkaiaawMcaaiabdsgaKjabdsha0bGaayzFaaaaaiaaxMaacaWLjaWaaeWaaeaaieaacqGFbbqqcqaIXaqmcqaI1aqnaiaawIcacaGLPaaaaaa@B7BB@

By the fact *E*{*V*(*x*(∞))} ≥ 0 and the in equality (A9), we get

E{∫0∞ZT(t)Z(t)dt}≤E{V(x(0))}+E{∫0∞(xT(t)CTCx(t)+(∂∂xV(x))TN(x)+12MT(x)∂2V(x)∂x2M(x)+14ρ2(∂∂xV(x))TH(x)HT(x)(∂∂xV(x)))dt}+ρ2E{∫0∞υT(t)υ(t)dt}     (A16)
 MathType@MTEF@5@5@+=feaafiart1ev1aaatCvAUfKttLearuWrP9MDH5MBPbIqV92AaeXatLxBI9gBaebbnrfifHhDYfgasaacH8akY=wiFfYdH8Gipec8Eeeu0xXdbba9frFj0=OqFfea0dXdd9vqai=hGuQ8kuc9pgc9s8qqaq=dirpe0xb9q8qiLsFr0=vr0=vr0dc8meaabaqaciaacaGaaeqabaqabeGadaaakeaafaqadeGabaaabaGaemyrau0aaiWaaeaadaWdXaqaaiabdQfaAnaaCaaaleqabaGaemivaqfaaOGaeiikaGIaemiDaqNaeiykaKIaemOwaOLaeiikaGIaemiDaqNaeiykaKIaemizaqMaemiDaqhaleaacqaIWaamaeaacqGHEisPa0Gaey4kIipaaOGaay5Eaiaaw2haaiabgsMiJkabdweafnaacmaabaGaemOvayLaeiikaGIaemiEaGNaeiikaGIaeGimaaJaeiykaKIaeiykaKcacaGL7bGaayzFaaGaey4kaSIaemyrau0aaiqaaeaadaWdXaqaamaabeaabaGaemiEaG3aaWbaaSqabeaacqWGubavaaGccqGGOaakcqWG0baDcqGGPaqkcqWGdbWqdaahaaWcbeqaaiabdsfaubaakiabdoeadjabdIha4jabcIcaOiabdsha0jabcMcaPiabgUcaRmaabmaabaWaaSaaaeaacqGHciITaeaacqGHciITcqWG4baEaaGaemOvayLaeiikaGIaemiEaGNaeiykaKcacaGLOaGaayzkaaWaaWbaaSqabeaacqWGubavaaGccqWGobGtcqGGOaakcqWG4baEcqGGPaqkaiaawIcaaaWcbaGaeGimaadabaGaeyOhIukaniabgUIiYdaakiaawUhaaaqaamaaciaabaWaaeGaaeaacqGHRaWkdaWcaaqaaiabigdaXaqaaiabikdaYaaacqWGnbqtdaahaaWcbeqaaiabdsfaubaakiabcIcaOiabdIha4jabcMcaPmaalaaabaGaeyOaIy7aaWbaaSqabeaacqaIYaGmaaGccqWGwbGvcqGGOaakcqWG4baEcqGGPaqkaeaacqGHciITcqWG4baEdaahaaWcbeqaaiabikdaYaaaaaGccqWGnbqtcqGGOaakcqWG4baEcqGGPaqkcqGHRaWkdaWcaaqaaiabigdaXaqaaiabisda0GGaciab=f8aYnaaCaaaleqabaGaeGOmaidaaaaakmaabmaabaWaaSaaaeaacqGHciITaeaacqGHciITcqWG4baEaaGaemOvayLaeiikaGIaemiEaGNaeiykaKcacaGLOaGaayzkaaWaaWbaaSqabeaacqWGubavaaGccqWGibascqGGOaakcqWG4baEcqGGPaqkcqWGibasdaahaaWcbeqaaiabdsfaubaakiabcIcaOiabdIha4jabcMcaPmaabmaabaWaaSaaaeaacqGHciITaeaacqGHciITcqWG4baEaaGaemOvayLaeiikaGIaemiEaGNaeiykaKcacaGLOaGaayzkaaaacaGLPaaacqWGKbazcqWG0baDaiaaw2haaiabgUcaRiab=f8aYnaaCaaaleqabaGaeGOmaidaaOGaemyrau0aaiWaaeaadaWdXaqaaiab=v8a1naaCaaaleqabaGaemivaqfaaOGaeiikaGIaemiDaqNaeiykaKIae8xXduNaeiikaGIaemiDaqNaeiykaKIaemizaqMaemiDaqhaleaacqaIWaamaeaacqGHEisPa0Gaey4kIipaaOGaay5Eaiaaw2haaaaacaWLjaGaaCzcamaabmaabaacbaGae4xqaeKaeGymaeJaeGOnaydacaGLOaGaayzkaaaaaa@D651@

By the inequality (19), we get

E{∫0∞ZT(t)Z(t)dt}≤E{V(x(0))}+ρ2E{∫0∞υT(t)υ(t)dt}
 MathType@MTEF@5@5@+=feaafiart1ev1aaatCvAUfKttLearuWrP9MDH5MBPbIqV92AaeXatLxBI9gBaebbnrfifHhDYfgasaacH8akY=wiFfYdH8Gipec8Eeeu0xXdbba9frFj0=OqFfea0dXdd9vqai=hGuQ8kuc9pgc9s8qqaq=dirpe0xb9q8qiLsFr0=vr0=vr0dc8meaabaqaciaacaGaaeqabaqabeGadaaakeaacqWGfbqrdaGadaqaamaapedabaGaemOwaO1aaWbaaSqabeaacqWGubavaaGccqGGOaakcqWG0baDcqGGPaqkcqWGAbGwcqGGOaakcqWG0baDcqGGPaqkcqWGKbazcqWG0baDaSqaaiabicdaWaqaaiabg6HiLcqdcqGHRiI8aaGccaGL7bGaayzFaaGaeyizImQaemyrau0aaiWaaeaacqWGwbGvcqGGOaakcqWG4baEcqGGOaakcqaIWaamcqGGPaqkcqGGPaqkaiaawUhacaGL9baacqGHRaWkcqaHbpGCdaahaaWcbeqaaiabikdaYaaakiabdweafnaacmaabaWaa8qmaeaacqaHfpqDdaahaaWcbeqaaiabdsfaubaaaeaacqaIWaamaeaacqGHEisPa0Gaey4kIipakmaabmaabaGaemiDaqhacaGLOaGaayzkaaGaeqyXdu3aaeWaaeaacqWG0baDaiaawIcacaGLPaaacqWGKbazcqWG0baDaiaawUhacaGL9baaaaa@669D@

or

‖Z(t)‖22≤E{V(x(0))}+ρ2‖υ(t)‖22
 MathType@MTEF@5@5@+=feaafiart1ev1aaatCvAUfKttLearuWrP9MDH5MBPbIqV92AaeXatLxBI9gBaebbnrfifHhDYfgasaacH8akY=wiFfYdH8Gipec8Eeeu0xXdbba9frFj0=OqFfea0dXdd9vqai=hGuQ8kuc9pgc9s8qqaq=dirpe0xb9q8qiLsFr0=vr0=vr0dc8meaabaqaciaacaGaaeqabaqabeGadaaakeaadaqbdaqaaiabdQfaAjabcIcaOiabdsha0jabcMcaPaGaayzcSlaawQa7amaaDaaaleaacqaIYaGmaeaacqaIYaGmaaGccqGHKjYOcqWGfbqrdaGadaqaaiabdAfawjabcIcaOiabdIha4jabcIcaOiabicdaWiabcMcaPiabcMcaPaGaay5Eaiaaw2haaiabgUcaRGGaciab=f8aYnaaCaaaleqabaGaeGOmaidaaOWaauWaaeaacqWFfpqDcqGGOaakcqWG0baDcqGGPaqkaiaawMa7caGLkWoadaqhaaWcbaGaeGOmaidabaGaeGOmaidaaaaa@5036@

### Appendix 5: details of computational simulations

The dynamic response of the typical genetic regulatory network in (22) is shown in Figure 3(a), where we can find that *X*_*e *_= [0.7339 0.7339 1 0.9283 0.9283]^*T *^is a stable equilibrium point of the genetic regulatory network. Since we are interested in the robust stability of the equilibrium under stochastic parameter perturbation, the origin should be shifted to the equilibrium. Then the genetic regulatory network in (22) under coordinate shift can be rewritten as follows

X˙′1=k1(X′3+1)(X5+0.9283)−1−k2(X′1+0.7339)2X′1(0)=−0.2339X˙′2=k3(X′1+0.7339)2−k4(X′2+0.7339)2X′2(0)=−0.2339X˙′3=k5(X′2+0.7339)−1−k6(X′2+0.7339)−1(X′3+1)2X′3(0)=−0.5X˙′4=k7(X′3+1)2(X′5+0.9283)−1−k8(X′4+0.9283)2X′4(0)=−0.4283X˙′5=k9(X′4+0.9283)−1−k10(X′5+0.9283)2X′5(0)=−0.4283     (A17)
 MathType@MTEF@5@5@+=feaafiart1ev1aaatCvAUfKttLearuWrP9MDH5MBPbIqV92AaeXatLxBI9gBaebbnrfifHhDYfgasaacH8akY=wiFfYdH8Gipec8Eeeu0xXdbba9frFj0=OqFfea0dXdd9vqai=hGuQ8kuc9pgc9s8qqaq=dirpe0xb9q8qiLsFr0=vr0=vr0dc8meaabaqaciaacaGaaeqabaqabeGadaaakeaafaqaaeqbcaaaaeaacuWGybawgaGagaqbamaaBaaaleaacqaIXaqmaeqaaOGaeyypa0Jaem4AaS2aaSbaaSqaaiabigdaXaqabaGccqGGOaakcuWGybawgaqbamaaBaaaleaacqaIZaWmaeqaaOGaey4kaSIaeGymaeJaeiykaKIaeiikaGIaemiwaG1aaSbaaSqaaiabiwda1aqabaGccqGHRaWkcqaIWaamcqGGUaGlcqaI5aqocqaIYaGmcqaI4aaocqaIZaWmcqGGPaqkdaahaaWcbeqaaiabgkHiTiabigdaXaaakiabgkHiTiabdUgaRnaaBaaaleaacqaIYaGmaeqaaOGaeiikaGIafmiwaGLbauaadaWgaaWcbaGaeGymaedabeaakiabgUcaRiabicdaWiabc6caUiabiEda3iabiodaZiabiodaZiabiMda5iabcMcaPmaaCaaaleqabaGaeGOmaidaaaGcbaGafmiwaGLbauaadaWgaaWcbaGaeGymaedabeaakiabcIcaOiabicdaWiabcMcaPiabg2da9iabgkHiTiabicdaWiabc6caUiabikdaYiabiodaZiabiodaZiabiMda5aqaaiqbdIfayzaacyaafaWaaSbaaSqaaiabikdaYaqabaGccqGH9aqpcqWGRbWAdaWgaaWcbaGaeG4mamdabeaakiabcIcaOiqbdIfayzaafaWaaSbaaSqaaiabigdaXaqabaGccqGHRaWkcqaIWaamcqGGUaGlcqaI3aWncqaIZaWmcqaIZaWmcqaI5aqocqGGPaqkdaahaaWcbeqaaiabikdaYaaakiabgkHiTiabdUgaRnaaBaaaleaacqaI0aanaeqaaOGaeiikaGIafmiwaGLbauaadaWgaaWcbaGaeGOmaidabeaakiabgUcaRiabicdaWiabc6caUiabiEda3iabiodaZiabiodaZiabiMda5iabcMcaPmaaCaaaleqabaGaeGOmaidaaaGcbaGafmiwaGLbauaadaWgaaWcbaGaeGOmaidabeaakiabcIcaOiabicdaWiabcMcaPiabg2da9iabgkHiTiabicdaWiabc6caUiabikdaYiabiodaZiabiodaZiabiMda5aqaaiqbdIfayzaacyaafaWaaSbaaSqaaiabiodaZaqabaGccqGH9aqpcqWGRbWAdaWgaaWcbaGaeGynaudabeaakiabcIcaOiqbdIfayzaafaWaaSbaaSqaaiabikdaYaqabaGccqGHRaWkcqaIWaamcqGGUaGlcqaI3aWncqaIZaWmcqaIZaWmcqaI5aqocqGGPaqkdaahaaWcbeqaaiabgkHiTiabigdaXaaakiabgkHiTiabdUgaRnaaBaaaleaacqaI2aGnaeqaaOGaeiikaGIafmiwaGLbauaadaWgaaWcbaGaeGOmaidabeaakiabgUcaRiabicdaWiabc6caUiabiEda3iabiodaZiabiodaZiabiMda5iabcMcaPmaaCaaaleqabaGaeyOeI0IaeGymaedaaOGaeiikaGIafmiwaGLbauaadaWgaaWcbaGaeG4mamdabeaakiabgUcaRiabigdaXiabcMcaPmaaCaaaleqabaGaeGOmaidaaaGcbaGafmiwaGLbauaadaWgaaWcbaGaeG4mamdabeaakiabcIcaOiabicdaWiabcMcaPiabg2da9iabgkHiTiabicdaWiabc6caUiabiwda1aqaaiqbdIfayzaacyaafaWaaSbaaSqaaiabisda0aqabaGccqGH9aqpcqWGRbWAdaWgaaWcbaGaeG4naCdabeaakiabcIcaOiqbdIfayzaafaWaaSbaaSqaaiabiodaZaqabaGccqGHRaWkcqaIXaqmcqGGPaqkdaahaaWcbeqaaiabikdaYaaakiabcIcaOiqbdIfayzaafaWaaSbaaSqaaiabiwda1aqabaGccqGHRaWkcqaIWaamcqGGUaGlcqaI5aqocqaIYaGmcqaI4aaocqaIZaWmcqGGPaqkdaahaaWcbeqaaiabgkHiTiabigdaXaaakiabgkHiTiabdUgaRnaaBaaaleaacqaI4aaoaeqaaOGaeiikaGIafmiwaGLbauaadaWgaaWcbaGaeGinaqdabeaakiabgUcaRiabicdaWiabc6caUiabiMda5iabikdaYiabiIda4iabiodaZiabcMcaPmaaCaaaleqabaGaeGOmaidaaaGcbaGafmiwaGLbauaadaWgaaWcbaGaeGinaqdabeaakiabcIcaOiabicdaWiabcMcaPiabg2da9iabgkHiTiabicdaWiabc6caUiabisda0iabikdaYiabiIda4iabiodaZaqaaiqbdIfayzaacyaafaWaaSbaaSqaaiabiwda1aqabaGccqGH9aqpcqWGRbWAdaWgaaWcbaGaeGyoaKdabeaakiabcIcaOiqbdIfayzaafaWaaSbaaSqaaiabisda0aqabaGccqGHRaWkcqaIWaamcqGGUaGlcqaI5aqocqaIYaGmcqaI4aaocqaIZaWmcqGGPaqkdaahaaWcbeqaaiabgkHiTiabigdaXaaakiabgkHiTiabdUgaRnaaBaaaleaacqaIXaqmcqaIWaamaeqaaOGaeiikaGIafmiwaGLbauaadaWgaaWcbaGaeGynaudabeaakiabgUcaRiabicdaWiabc6caUiabiMda5iabikdaYiabiIda4iabiodaZiabcMcaPmaaCaaaleqabaGaeGOmaidaaaGcbaGafmiwaGLbauaadaWgaaWcbaGaeGynaudabeaakiabcIcaOiabicdaWiabcMcaPiabg2da9iabgkHiTiabicdaWiabc6caUiabisda0iabikdaYiabiIda4iabiodaZaaacaWLjaGaaCzcamaabmaabaacbaGae8xqaeKaeGymaeJaeG4naCdacaGLOaGaayzkaaaaaa@2F17@

and the dynamic response of the genetic regulatory network under coordinate shift is shown in Figure 3(b).

Suppose the kinetic parameters *k*_*i*_, *i *= 1,…,10, suffer some stochastic perturbations *k*_*i *_+ Δ*k*_*i*_, *i *= 1, …,10, respectively, where Δ*k*_1 _= 1*n*(*t*), Δ*k*_2 _= 2*n*(*t*), Δ*k*_3 _= -2*n*(*t*), Δ*k*_4 _= -0.3*n*(*t*), Δ*k*_5 _= 0.8 *n*(*t*), Δ*k*_6 _= 1.4*n*(*t*), Δ*k*_7 _= 1.2(*t*), Δ*k*_8 _= 2*n*(*t*), Δ*k*_9 _= -1.8*n*(*t*) and *n*(*t*) denotes the standard zero mean white Gaussian noise with unit variance. Then the genetic regulatory network under such perturbations becomes the following nonlinear stochastic system

dX1=(5X3X5−1−10X12)dt+(X3X5−1−2X12)dωX1(0)=0.5dX2=(10X12−10X22)dt+(−2X12+0.3X22)dωX2(0)=0.5dX3=(10X2−1−10X2−1X32)dt+(0.8X2−1−1.4X2−1X32)dωX3(0)=0.5dX4=(8X32X5−1−10X42)dt+(1.2X32X5−1−2X42)dωX4(0)=0.5dX5=(10X42−10X52)dt−1.8X52dωX5(0)=0.5     (A18)
 MathType@MTEF@5@5@+=feaafiart1ev1aaatCvAUfKttLearuWrP9MDH5MBPbIqV92AaeXatLxBI9gBaebbnrfifHhDYfgasaacH8akY=wiFfYdH8Gipec8Eeeu0xXdbba9frFj0=OqFfea0dXdd9vqai=hGuQ8kuc9pgc9s8qqaq=dirpe0xb9q8qiLsFr0=vr0=vr0dc8meaabaqaciaacaGaaeqabaqabeGadaaakeaafaqaaeqbcaaaaeaacqWGKbazcqWGybawdaWgaaWcbaGaeGymaedabeaakiabg2da9maabmaabaGaeGynauJaemiwaG1aaSbaaSqaaiabiodaZaqabaGccqWGybawdaqhaaWcbaGaeGynaudabaGaeyOeI0IaeGymaedaaOGaeyOeI0IaeGymaeJaeGimaaJaemiwaG1aa0baaSqaaiabigdaXaqaaiabikdaYaaaaOGaayjkaiaawMcaaiabdsgaKjabdsha0jabgUcaRmaabmaabaGaemiwaG1aaSbaaSqaaiabiodaZaqabaGccqWGybawdaqhaaWcbaGaeGynaudabaGaeyOeI0IaeGymaedaaOGaeyOeI0IaeGOmaiJaemiwaG1aa0baaSqaaiabigdaXaqaaiabikdaYaaaaOGaayjkaiaawMcaaiabdsgaKHGaciab=L8a3bqaaiabdIfaynaaBaaaleaacqaIXaqmaeqaaOGaeiikaGIaeGimaaJaeiykaKIaeyypa0JaeGimaaJaeiOla4IaeGynaudabaGaemizaqMaemiwaG1aaSbaaSqaaiabikdaYaqabaGccqGH9aqpdaqadaqaaiabigdaXiabicdaWiabdIfaynaaDaaaleaacqaIXaqmaeaacqaIYaGmaaGccqGHsislcqaIXaqmcqaIWaamcqWGybawdaqhaaWcbaGaeGOmaidabaGaeGOmaidaaaGccaGLOaGaayzkaaGaemizaqMaemiDaqNaey4kaSYaaeWaaeaacqGHsislcqaIYaGmcqWGybawdaqhaaWcbaGaeGymaedabaGaeGOmaidaaOGaey4kaSIaeGimaaJaeiOla4IaeG4mamJaemiwaG1aa0baaSqaaiabikdaYaqaaiabikdaYaaaaOGaayjkaiaawMcaaiabdsgaKjab=L8a3bqaaiabdIfaynaaBaaaleaacqaIYaGmaeqaaOGaeiikaGIaeGimaaJaeiykaKIaeyypa0JaeGimaaJaeiOla4IaeGynaudabaGaemizaqMaemiwaG1aaSbaaSqaaiabiodaZaqabaGccqGH9aqpdaqadaqaaiabigdaXiabicdaWiabdIfaynaaDaaaleaacqaIYaGmaeaacqGHsislcqaIXaqmaaGccqGHsislcqaIXaqmcqaIWaamcqWGybawdaqhaaWcbaGaeGOmaidabaGaeyOeI0IaeGymaedaaOGaemiwaG1aa0baaSqaaiabiodaZaqaaiabikdaYaaaaOGaayjkaiaawMcaaiabdsgaKjabdsha0jabgUcaRmaabmaabaGaeGimaaJaeiOla4IaeGioaGJaemiwaG1aa0baaSqaaiabikdaYaqaaiabgkHiTiabigdaXaaakiabgkHiTiabigdaXiabc6caUiabisda0iabdIfaynaaDaaaleaacqaIYaGmaeaacqGHsislcqaIXaqmaaGccqWGybawdaqhaaWcbaGaeG4mamdabaGaeGOmaidaaaGccaGLOaGaayzkaaGaemizaqMae8xYdChabaGaemiwaG1aaSbaaSqaaiabiodaZaqabaGccqGGOaakcqaIWaamcqGGPaqkcqGH9aqpcqaIWaamcqGGUaGlcqaI1aqnaeaacqWGKbazcqWGybawdaWgaaWcbaGaeGinaqdabeaakiabg2da9maabmaabaGaeGioaGJaemiwaG1aa0baaSqaaiabiodaZaqaaiabikdaYaaakiabdIfaynaaDaaaleaacqaI1aqnaeaacqGHsislcqaIXaqmaaGccqGHsislcqaIXaqmcqaIWaamcqWGybawdaqhaaWcbaGaeGinaqdabaGaeGOmaidaaaGccaGLOaGaayzkaaGaemizaqMaemiDaqNaey4kaSYaaeWaaeaacqaIXaqmcqGGUaGlcqaIYaGmcqWGybawdaqhaaWcbaGaeG4mamdabaGaeGOmaidaaOGaemiwaG1aa0baaSqaaiabiwda1aqaaiabgkHiTiabigdaXaaakiabgkHiTiabikdaYiabdIfaynaaDaaaleaacqaI0aanaeaacqaIYaGmaaaakiaawIcacaGLPaaacqWGKbazcqWFjpWDaeaacqWGybawdaWgaaWcbaGaeGinaqdabeaakiabcIcaOiabicdaWiabcMcaPiabg2da9iabicdaWiabc6caUiabiwda1aqaaiabdsgaKjabdIfaynaaBaaaleaacqaI1aqnaeqaaOGaeyypa0ZaaeWaaeaacqaIXaqmcqaIWaamcqWGybawdaqhaaWcbaGaeGinaqdabaGaeGOmaidaaOGaeyOeI0IaeGymaeJaeGimaaJaemiwaG1aa0baaSqaaiabiwda1aqaaiabikdaYaaaaOGaayjkaiaawMcaaiabdsgaKjabdsha0jabgkHiTiabigdaXiabc6caUiabiIda4iabdIfaynaaDaaaleaacqaI1aqnaeaacqaIYaGmaaGccqWGKbazcqWFjpWDaeaacqWGybawdaWgaaWcbaGaeGynaudabeaakiabcIcaOiabicdaWiabcMcaPiabg2da9iabicdaWiabc6caUiabiwda1aaacaWLjaGaaCzcamaabmaabaacbaGae4xqaeKaeGymaeJaeGioaGdacaGLOaGaayzkaaaaaa@29AD@

We can rewrite the genetic regulatory network under such perturbations by coordinate shift as (6) as follows

dX′1=(5(X′3+1)(X′5+0.9283)−1−10(X′1+0.7339)2)dt+((X′3+1)(X′5+0.9283)−1−2(X′1+0.7339)2)dωdX′2=(10(X′1+0.7339)2−10(X′2+0.7339)2)dt+(−2(X′1+0.7339)2+0.3(X′2+0.7339)2)dωdX′3=(10(X′2+0.7339)−1−10(X′2+0.7339)−1(X′3+1)2)dt+(0.8(X′2+0.7339)−1−1.4(X′2+0.7339)−1(X′3+1)2)dωdX′4=(8(X′3+1)2(X′5+0.9283)−1−10(X′4+0.9283)2)dt+(1.2(X′3+1)2(X′5+0.9283)−1−2(X′4+0.9283)2)dωdX′5=(10(X′4+0.9283)2−10(X′5+0.9283)2)dt−1.8(X′4+0.9283)2dωX′1(0)=−0.2339,X′2(0)=−0.2339,X′3(0)=−0.5,X′4(0)=−0.4283,X′5(0)=−0.4283     (A19)
 MathType@MTEF@5@5@+=feaafiart1ev1aaatCvAUfKttLearuWrP9MDH5MBPbIqV92AaeXatLxBI9gBaebbnrfifHhDYfgasaacH8akY=wiFfYdH8Gipec8Eeeu0xXdbba9frFj0=OqFfea0dXdd9vqai=hGuQ8kuc9pgc9s8qqaq=dirpe0xb9q8qiLsFr0=vr0=vr0dc8meaabaqaciaacaGaaeqabaqabeGadaaakqaabeqaaiabdsgaKjqbdIfayzaafaWaaSbaaSqaaiabigdaXaqabaGccqGH9aqpdaqadaqaaiabiwda1maabmaabaGafmiwaGLbauaadaWgaaWcbaGaeG4mamdabeaakiabgUcaRiabigdaXaGaayjkaiaawMcaamaabmaabaGafmiwaGLbauaadaWgaaWcbaGaeGynaudabeaakiabgUcaRiabicdaWiabc6caUiabiMda5iabikdaYiabiIda4iabiodaZaGaayjkaiaawMcaamaaCaaaleqabaGaeyOeI0IaeGymaedaaOGaeyOeI0IaeGymaeJaeGimaaZaaeWaaeaacuWGybawgaqbamaaBaaaleaacqaIXaqmaeqaaOGaey4kaSIaeGimaaJaeiOla4IaeG4naCJaeG4mamJaeG4mamJaeGyoaKdacaGLOaGaayzkaaWaaWbaaSqabeaacqaIYaGmaaaakiaawIcacaGLPaaacqWGKbazcqWG0baDcqGHRaWkdaqadaqaamaabmaabaGafmiwaGLbauaadaWgaaWcbaGaeG4mamdabeaakiabgUcaRiabigdaXaGaayjkaiaawMcaamaabmaabaGafmiwaGLbauaadaWgaaWcbaGaeGynaudabeaakiabgUcaRiabicdaWiabc6caUiabiMda5iabikdaYiabiIda4iabiodaZaGaayjkaiaawMcaamaaCaaaleqabaGaeyOeI0IaeGymaedaaOGaeyOeI0IaeGOmaiZaaeWaaeaacuWGybawgaqbamaaBaaaleaacqaIXaqmaeqaaOGaey4kaSIaeGimaaJaeiOla4IaeG4naCJaeG4mamJaeG4mamJaeGyoaKdacaGLOaGaayzkaaWaaWbaaSqabeaacqaIYaGmaaaakiaawIcacaGLPaaacqWGKbaziiGacqWFjpWDaeaacqWGKbazcuWGybawgaqbamaaBaaaleaacqaIYaGmaeqaaOGaeyypa0ZaaeWaaeaacqaIXaqmcqaIWaamdaqadaqaaiqbdIfayzaafaWaaSbaaSqaaiabigdaXaqabaGccqGHRaWkcqaIWaamcqGGUaGlcqaI3aWncqaIZaWmcqaIZaWmcqaI5aqoaiaawIcacaGLPaaadaahaaWcbeqaaiabikdaYaaakiabgkHiTiabigdaXiabicdaWmaabmaabaGafmiwaGLbauaadaWgaaWcbaGaeGOmaidabeaakiabgUcaRiabicdaWiabc6caUiabiEda3iabiodaZiabiodaZiabiMda5aGaayjkaiaawMcaamaaCaaaleqabaGaeGOmaidaaaGccaGLOaGaayzkaaGaemizaqMaemiDaqNaey4kaSYaaeWaaeaacqGHsislcqaIYaGmdaqadaqaaiqbdIfayzaafaWaaSbaaSqaaiabigdaXaqabaGccqGHRaWkcqaIWaamcqGGUaGlcqaI3aWncqaIZaWmcqaIZaWmcqaI5aqoaiaawIcacaGLPaaadaahaaWcbeqaaiabikdaYaaakiabgUcaRiabicdaWiabc6caUiabiodaZmaabmaabaGafmiwaGLbauaadaWgaaWcbaGaeGOmaidabeaakiabgUcaRiabicdaWiabc6caUiabiEda3iabiodaZiabiodaZiabiMda5aGaayjkaiaawMcaamaaCaaaleqabaGaeGOmaidaaaGccaGLOaGaayzkaaGaemizaqMae8xYdChabaGaemizaqMafmiwaGLbauaadaWgaaWcbaGaeG4mamdabeaakiabg2da9maabmaabaGaeGymaeJaeGimaaZaaeWaaeaacuWGybawgaqbamaaBaaaleaacqaIYaGmaeqaaOGaey4kaSIaeGimaaJaeiOla4IaeG4naCJaeG4mamJaeG4mamJaeGyoaKdacaGLOaGaayzkaaWaaWbaaSqabeaacqGHsislcqaIXaqmaaGccqGHsislcqaIXaqmcqaIWaamdaqadaqaaiqbdIfayzaafaWaaSbaaSqaaiabikdaYaqabaGccqGHRaWkcqaIWaamcqGGUaGlcqaI3aWncqaIZaWmcqaIZaWmcqaI5aqoaiaawIcacaGLPaaadaahaaWcbeqaaiabgkHiTiabigdaXaaakmaabmaabaGafmiwaGLbauaadaWgaaWcbaGaeG4mamdabeaakiabgUcaRiabigdaXaGaayjkaiaawMcaamaaCaaaleqabaGaeGOmaidaaaGccaGLOaGaayzkaaGaemizaqMaemiDaqNaey4kaSYaaeWaaeaacqaIWaamcqGGUaGlcqaI4aaodaqadaqaaiqbdIfayzaafaWaaSbaaSqaaiabikdaYaqabaGccqGHRaWkcqaIWaamcqGGUaGlcqaI3aWncqaIZaWmcqaIZaWmcqaI5aqoaiaawIcacaGLPaaadaahaaWcbeqaaiabgkHiTiabigdaXaaakiabgkHiTiabigdaXiabc6caUiabisda0maabmaabaGafmiwaGLbauaadaWgaaWcbaGaeGOmaidabeaakiabgUcaRiabicdaWiabc6caUiabiEda3iabiodaZiabiodaZiabiMda5aGaayjkaiaawMcaamaaCaaaleqabaGaeyOeI0IaeGymaedaaOWaaeWaaeaacuWGybawgaqbamaaBaaaleaacqaIZaWmaeqaaOGaey4kaSIaeGymaedacaGLOaGaayzkaaWaaWbaaSqabeaacqaIYaGmaaaakiaawIcacaGLPaaacqWGKbazcqWFjpWDaeaacqWGKbazcuWGybawgaqbamaaBaaaleaacqaI0aanaeqaaOGaeyypa0ZaaeWaaeaacqaI4aaodaqadaqaaiqbdIfayzaafaWaaSbaaSqaaiabiodaZaqabaGccqGHRaWkcqaIXaqmaiaawIcacaGLPaaadaahaaWcbeqaaiabikdaYaaakmaabmaabaGafmiwaGLbauaadaWgaaWcbaGaeGynaudabeaakiabgUcaRiabicdaWiabc6caUiabiMda5iabikdaYiabiIda4iabiodaZaGaayjkaiaawMcaamaaCaaaleqabaGaeyOeI0IaeGymaedaaOGaeyOeI0IaeGymaeJaeGimaaZaaeWaaeaacuWGybawgaqbamaaBaaaleaacqaI0aanaeqaaOGaey4kaSIaeGimaaJaeiOla4IaeGyoaKJaeGOmaiJaeGioaGJaeG4mamdacaGLOaGaayzkaaWaaWbaaSqabeaacqaIYaGmaaaakiaawIcacaGLPaaacqWGKbazcqWG0baDcqGHRaWkdaqadaqaaiabigdaXiabc6caUiabikdaYmaabmaabaGafmiwaGLbauaadaWgaaWcbaGaeG4mamdabeaakiabgUcaRiabigdaXaGaayjkaiaawMcaamaaCaaaleqabaGaeGOmaidaaOWaaeWaaeaacuWGybawgaqbamaaBaaaleaacqaI1aqnaeqaaOGaey4kaSIaeGimaaJaeiOla4IaeGyoaKJaeGOmaiJaeGioaGJaeG4mamdacaGLOaGaayzkaaWaaWbaaSqabeaacqGHsislcqaIXaqmaaGccqGHsislcqaIYaGmdaqadaqaaiqbdIfayzaafaWaaSbaaSqaaiabisda0aqabaGccqGHRaWkcqaIWaamcqGGUaGlcqaI5aqocqaIYaGmcqaI4aaocqaIZaWmaiaawIcacaGLPaaadaahaaWcbeqaaiabikdaYaaaaOGaayjkaiaawMcaaiabdsgaKjab=L8a3bqaaiabdsgaKjqbdIfayzaafaWaaSbaaSqaaiabiwda1aqabaGccqGH9aqpdaqadaqaaiabigdaXiabicdaWmaabmaabaGafmiwaGLbauaadaWgaaWcbaGaeGinaqdabeaakiabgUcaRiabicdaWiabc6caUiabiMda5iabikdaYiabiIda4iabiodaZaGaayjkaiaawMcaamaaCaaaleqabaGaeGOmaidaaOGaeyOeI0IaeGymaeJaeGimaaZaaeWaaeaacuWGybawgaqbamaaBaaaleaacqaI1aqnaeqaaOGaey4kaSIaeGimaaJaeiOla4IaeGyoaKJaeGOmaiJaeGioaGJaeG4mamdacaGLOaGaayzkaaWaaWbaaSqabeaacqaIYaGmaaaakiaawIcacaGLPaaacqWGKbazcqWG0baDcqGHsislcqaIXaqmcqGGUaGlcqaI4aaodaqadaqaaiqbdIfayzaafaWaaSbaaSqaaiabisda0aqabaGccqGHRaWkcqaIWaamcqGGUaGlcqaI5aqocqaIYaGmcqaI4aaocqaIZaWmaiaawIcacaGLPaaadaahaaWcbeqaaiabikdaYaaakiabdsgaKjab=L8a3bqaaiqbdIfayzaafaWaaSbaaSqaaiabigdaXaqabaGccqGGOaakcqaIWaamcqGGPaqkcqGH9aqpcqGHsislcqaIWaamcqGGUaGlcqaIYaGmcqaIZaWmcqaIZaWmcqaI5aqocqGGSaalcuWGybawgaqbamaaBaaaleaacqaIYaGmaeqaaOGaeiikaGIaeGimaaJaeiykaKIaeyypa0JaeyOeI0IaeGimaaJaeiOla4IaeGOmaiJaeG4mamJaeG4mamJaeGyoaKJaeiilaWIafmiwaGLbauaadaWgaaWcbaGaeG4mamdabeaakiabcIcaOiabicdaWiabcMcaPiabg2da9iabgkHiTiabicdaWiabc6caUiabiwda1iabcYcaSiqbdIfayzaafaWaaSbaaSqaaiabisda0aqabaGccqGGOaakcqaIWaamcqGGPaqkcqGH9aqpcqGHsislcqaIWaamcqGGUaGlcqaI0aancqaIYaGmcqaI4aaocqaIZaWmcqGGSaalcuWGybawgaqbamaaBaaaleaacqaI1aqnaeqaaOGaeiikaGIaeGimaaJaeiykaKIaeyypa0JaeyOeI0IaeGimaaJaeiOla4IaeGinaqJaeGOmaiJaeGioaGJaeG4mamJaaCzcaiaaxMaacqGGOaakieaacqGFbbqqcqaIXaqmcqaI5aqocqGGPaqkaaaa@F285@

In the perturbative case, we can use global linearization method and solve the LMIs in (21), Then the solution *P *of the LMIs in (21) is found as

P=[0.381960.076187−0.024633−0.1377−0.0338690.0761870.21315−0.01921−0.026898−0.033226−0.024633−0.019210.125030.0021901−0.065039−0.1377−0.0268980.00219010.259560.025702−0.033869−0.033226−0.0650390.0257020.18759]
 MathType@MTEF@5@5@+=feaafiart1ev1aaatCvAUfKttLearuWrP9MDH5MBPbIqV92AaeXatLxBI9gBaebbnrfifHhDYfgasaacH8akY=wiFfYdH8Gipec8Eeeu0xXdbba9frFj0=OqFfea0dXdd9vqai=hGuQ8kuc9pgc9s8qqaq=dirpe0xb9q8qiLsFr0=vr0=vr0dc8meaabaqaciaacaGaaeqabaqabeGadaaakeaacqWGqbaucqGH9aqpdaWadaqaauaabeqafuaaaaaabaGaeGimaaJaeiOla4IaeG4mamJaeGioaGJaeGymaeJaeGyoaKJaeGOnaydabaGaeGimaaJaeiOla4IaeGimaaJaeG4naCJaeGOnayJaeGymaeJaeGioaGJaeG4naCdabaGaeyOeI0IaeGimaaJaeiOla4IaeGimaaJaeGOmaiJaeGinaqJaeGOnayJaeG4mamJaeG4mamdabaGaeyOeI0IaeGimaaJaeiOla4IaeGymaeJaeG4mamJaeG4naCJaeG4naCdabaGaeyOeI0IaeGimaaJaeiOla4IaeGimaaJaeG4mamJaeG4mamJaeGioaGJaeGOnayJaeGyoaKdabaGaeGimaaJaeiOla4IaeGimaaJaeG4naCJaeGOnayJaeGymaeJaeGioaGJaeG4naCdabaGaeGimaaJaeiOla4IaeGOmaiJaeGymaeJaeG4mamJaeGymaeJaeGynaudabaGaeyOeI0IaeGimaaJaeiOla4IaeGimaaJaeGymaeJaeGyoaKJaeGOmaiJaeGymaedabaGaeyOeI0IaeGimaaJaeiOla4IaeGimaaJaeGOmaiJaeGOnayJaeGioaGJaeGyoaKJaeGioaGdabaGaeyOeI0IaeGimaaJaeiOla4IaeGimaaJaeG4mamJaeG4mamJaeGOmaiJaeGOmaiJaeGOnaydabaGaeyOeI0IaeGimaaJaeiOla4IaeGimaaJaeGOmaiJaeGinaqJaeGOnayJaeG4mamJaeG4mamdabaGaeyOeI0IaeGimaaJaeiOla4IaeGimaaJaeGymaeJaeGyoaKJaeGOmaiJaeGymaedabaGaeGimaaJaeiOla4IaeGymaeJaeGOmaiJaeGynauJaeGimaaJaeG4mamdabaGaeGimaaJaeiOla4IaeGimaaJaeGimaaJaeGOmaiJaeGymaeJaeGyoaKJaeGimaaJaeGymaedabaGaeyOeI0IaeGimaaJaeiOla4IaeGimaaJaeGOnayJaeGynauJaeGimaaJaeG4mamJaeGyoaKdabaGaeyOeI0IaeGimaaJaeiOla4IaeGymaeJaeG4mamJaeG4naCJaeG4naCdabaGaeyOeI0IaeGimaaJaeiOla4IaeGimaaJaeGOmaiJaeGOnayJaeGioaGJaeGyoaKJaeGioaGdabaGaeGimaaJaeiOla4IaeGimaaJaeGimaaJaeGOmaiJaeGymaeJaeGyoaKJaeGimaaJaeGymaedabaGaeGimaaJaeiOla4IaeGOmaiJaeGynauJaeGyoaKJaeGynauJaeGOnaydabaGaeGimaaJaeiOla4IaeGimaaJaeGOmaiJaeGynauJaeG4naCJaeGimaaJaeGOmaidabaGaeyOeI0IaeGimaaJaeiOla4IaeGimaaJaeG4mamJaeG4mamJaeGioaGJaeGOnayJaeGyoaKdabaGaeyOeI0IaeGimaaJaeiOla4IaeGimaaJaeG4mamJaeG4mamJaeGOmaiJaeGOmaiJaeGOnaydabaGaeyOeI0IaeGimaaJaeiOla4IaeGimaaJaeGOnayJaeGynauJaeGimaaJaeG4mamJaeGyoaKdabaGaeGimaaJaeiOla4IaeGimaaJaeGOmaiJaeGynauJaeG4naCJaeGimaaJaeGOmaidabaGaeGimaaJaeiOla4IaeGymaeJaeGioaGJaeG4naCJaeGynauJaeGyoaKdaaaGaay5waiaaw2faaaaa@F29C@

where the eigenvalues of *P *are 0.074151, 0.15722, 0.20491, 0.22885, 0.50216, which are all positive, so the stochastic system (A19) is stable in probability at *x' *= 0 under this intrinsic noise. The dynamic responses of the perturbed stochastic systems in (A18) and (A19) are shown in Figure 3(c) and Figure 3(d), respectively, which confirms the conclusion of our proposed computational method of robust stability.

If the kinetic parameters suffer the following stochastic perturbations Δ*k*_1 _= 4*n*(*t*), Δ*k*_2 _= -2*n*(*t*), Δ*k*_3 _= 6*n*(*t*), Δ*k*_4 _= -7*n*(*t*), Δ*k*_5 _= 5*n*(*t*), Δ*k*_6 _= 2*n*(*t*), Δ*k*_7 _= 6*n*(*t*), Δ*k*_8 _= 2.5*n*(*t*) Δ*k*_9 _= 8*n*(*t*), Δ*k*_10 _= - 6*n*(*t*) the network of the stochastic parameter pertubative case becomes the following nonlinear stochastic system

dX1=(5X3X5−1−10X12)dt+(4X3X5−1−2X12)dωX1(0)=0.5dX2=(10X12−10X22)dt+(6X12+7X22)dωX2(0)=0.5dX3=(10X2−1−10X2−1X32)dt+(5X2−1−2X2−1X32)dωX3(0)=0.5dX4=(8X32X5−1−10X42)dt+(6X32X5−1−2.5X42)dωX4(0)=0.5dX5=(10X42−10X52)dt+(8X42+6X52)dωX5(0)=0.5     (A20)
 MathType@MTEF@5@5@+=feaafiart1ev1aaatCvAUfKttLearuWrP9MDH5MBPbIqV92AaeXatLxBI9gBaebbnrfifHhDYfgasaacH8akY=wiFfYdH8Gipec8Eeeu0xXdbba9frFj0=OqFfea0dXdd9vqai=hGuQ8kuc9pgc9s8qqaq=dirpe0xb9q8qiLsFr0=vr0=vr0dc8meaabaqaciaacaGaaeqabaqabeGadaaakeaafaqaaeqbcaaaaeaacqWGKbazcqWGybawdaWgaaWcbaGaeGymaedabeaakiabg2da9maabmaabaGaeGynauJaemiwaG1aaSbaaSqaaiabiodaZaqabaGccqWGybawdaqhaaWcbaGaeGynaudabaGaeyOeI0IaeGymaedaaOGaeyOeI0IaeGymaeJaeGimaaJaemiwaG1aa0baaSqaaiabigdaXaqaaiabikdaYaaaaOGaayjkaiaawMcaaiabdsgaKjabdsha0jabgUcaRmaabmaabaGaeGinaqJaemiwaG1aaSbaaSqaaiabiodaZaqabaGccqWGybawdaqhaaWcbaGaeGynaudabaGaeyOeI0IaeGymaedaaOGaeyOeI0IaeGOmaiJaemiwaG1aa0baaSqaaiabigdaXaqaaiabikdaYaaaaOGaayjkaiaawMcaaiabdsgaKHGaciab=L8a3bqaaiabdIfaynaaBaaaleaacqaIXaqmaeqaaOGaeiikaGIaeGimaaJaeiykaKIaeyypa0JaeGimaaJaeiOla4IaeGynaudabaGaemizaqMaemiwaG1aaSbaaSqaaiabikdaYaqabaGccqGH9aqpdaqadaqaaiabigdaXiabicdaWiabdIfaynaaDaaaleaacqaIXaqmaeaacqaIYaGmaaGccqGHsislcqaIXaqmcqaIWaamcqWGybawdaqhaaWcbaGaeGOmaidabaGaeGOmaidaaaGccaGLOaGaayzkaaGaemizaqMaemiDaqNaey4kaSYaaeWaaeaacqaI2aGncqWGybawdaqhaaWcbaGaeGymaedabaGaeGOmaidaaOGaey4kaSIaeG4naCJaemiwaG1aa0baaSqaaiabikdaYaqaaiabikdaYaaaaOGaayjkaiaawMcaaiabdsgaKjab=L8a3bqaaiabdIfaynaaBaaaleaacqaIYaGmaeqaaOGaeiikaGIaeGimaaJaeiykaKIaeyypa0JaeGimaaJaeiOla4IaeGynaudabaGaemizaqMaemiwaG1aaSbaaSqaaiabiodaZaqabaGccqGH9aqpdaqadaqaaiabigdaXiabicdaWiabdIfaynaaDaaaleaacqaIYaGmaeaacqGHsislcqaIXaqmaaGccqGHsislcqaIXaqmcqaIWaamcqWGybawdaqhaaWcbaGaeGOmaidabaGaeyOeI0IaeGymaedaaOGaemiwaG1aa0baaSqaaiabiodaZaqaaiabikdaYaaaaOGaayjkaiaawMcaaiabdsgaKjabdsha0jabgUcaRmaabmaabaGaeGynauJaemiwaG1aa0baaSqaaiabikdaYaqaaiabgkHiTiabigdaXaaakiabgkHiTiabikdaYiabdIfaynaaDaaaleaacqaIYaGmaeaacqGHsislcqaIXaqmaaGccqWGybawdaqhaaWcbaGaeG4mamdabaGaeGOmaidaaaGccaGLOaGaayzkaaGaemizaqMae8xYdChabaGaemiwaG1aaSbaaSqaaiabiodaZaqabaGccqGGOaakcqaIWaamcqGGPaqkcqGH9aqpcqaIWaamcqGGUaGlcqaI1aqnaeaacqWGKbazcqWGybawdaWgaaWcbaGaeGinaqdabeaakiabg2da9maabmaabaGaeGioaGJaemiwaG1aa0baaSqaaiabiodaZaqaaiabikdaYaaakiabdIfaynaaDaaaleaacqaI1aqnaeaacqGHsislcqaIXaqmaaGccqGHsislcqaIXaqmcqaIWaamcqWGybawdaqhaaWcbaGaeGinaqdabaGaeGOmaidaaaGccaGLOaGaayzkaaGaemizaqMaemiDaqNaey4kaSYaaeWaaeaacqaI2aGncqWGybawdaqhaaWcbaGaeG4mamdabaGaeGOmaidaaOGaemiwaG1aa0baaSqaaiabiwda1aqaaiabgkHiTiabigdaXaaakiabgkHiTiabikdaYiabc6caUiabiwda1iabdIfaynaaDaaaleaacqaI0aanaeaacqaIYaGmaaaakiaawIcacaGLPaaacqWGKbazcqWFjpWDaeaacqWGybawdaWgaaWcbaGaeGinaqdabeaakiabcIcaOiabicdaWiabcMcaPiabg2da9iabicdaWiabc6caUiabiwda1aqaaiabdsgaKjabdIfaynaaBaaaleaacqaI1aqnaeqaaOGaeyypa0ZaaeWaaeaacqaIXaqmcqaIWaamcqWGybawdaqhaaWcbaGaeGinaqdabaGaeGOmaidaaOGaeyOeI0IaeGymaeJaeGimaaJaemiwaG1aa0baaSqaaiabiwda1aqaaiabikdaYaaaaOGaayjkaiaawMcaaiabdsgaKjabdsha0jabgUcaRmaabmaabaGaeGioaGJaemiwaG1aa0baaSqaaiabisda0aqaaiabikdaYaaakiabgUcaRiabiAda2iabdIfaynaaDaaaleaacqaI1aqnaeaacqaIYaGmaaaakiaawIcacaGLPaaacqWGKbazcqWFjpWDaeaacqWGybawdaWgaaWcbaGaeGynaudabeaakiabcIcaOiabicdaWiabcMcaPiabg2da9iabicdaWiabc6caUiabiwda1aaacaWLjaGaaCzcamaabmaabaacbaGae4xqaeKaeGOmaiJaeGimaadacaGLOaGaayzkaaaaaa@2924@

We again rewrite the genetic regulatory network under such perturbations by coordinate shift as follows

dX′1=(5(X′3+1)(X′5+0.9283)−1−10(X′1+0.7339)2)dt+(4(X′3+1)(X′5+0.9283)−1+2(X′1+0.7339)2)dωdX′2=(10(X′1+0.7339)2−10(X′2+0.7339)2)dt+(6(X′1+0.7339)2+7(X′2+0.7339)2)dωdX′3=(10(X′2+0.7339)−1−10(X′2+0.7339)−1(X′3+1)2)dt+(5(X′2+0.7339)−1−2(X′2+0.7339)−1(X′3+1)2)dωdX′4=(8(X′3+1)2(X′5+0.9283)−1−10(X′4+0.9283)2)dt+(6(X′3+1)2(X′5+0.9283)−1−2.5(X′4+0.9283)2)dωdX′5=(10(X′4+0.9283)2−10(X′5+0.9283)2)dt+(8(X′4+0.9283)2+6(X′5+0.9283)2)dωX′1(0)=−0.2339,X′2(0)=−0.2339,X′3(0)=−0.5,X′4(0)=−0.4283,X′5(0)=−0.4283     (A21)
 MathType@MTEF@5@5@+=feaafiart1ev1aaatCvAUfKttLearuWrP9MDH5MBPbIqV92AaeXatLxBI9gBaebbnrfifHhDYfgasaacH8akY=wiFfYdH8Gipec8Eeeu0xXdbba9frFj0=OqFfea0dXdd9vqai=hGuQ8kuc9pgc9s8qqaq=dirpe0xb9q8qiLsFr0=vr0=vr0dc8meaabaqaciaacaGaaeqabaqabeGadaaakqaabeqaaiabdsgaKjqbdIfayzaafaWaaSbaaSqaaiabigdaXaqabaGccqGH9aqpdaqadaqaaiabiwda1maabmaabaGafmiwaGLbauaadaWgaaWcbaGaeG4mamdabeaakiabgUcaRiabigdaXaGaayjkaiaawMcaamaabmaabaGafmiwaGLbauaadaWgaaWcbaGaeGynaudabeaakiabgUcaRiabicdaWiabc6caUiabiMda5iabikdaYiabiIda4iabiodaZaGaayjkaiaawMcaamaaCaaaleqabaGaeyOeI0IaeGymaedaaOGaeyOeI0IaeGymaeJaeGimaaZaaeWaaeaacuWGybawgaqbamaaBaaaleaacqaIXaqmaeqaaOGaey4kaSIaeGimaaJaeiOla4IaeG4naCJaeG4mamJaeG4mamJaeGyoaKdacaGLOaGaayzkaaWaaWbaaSqabeaacqaIYaGmaaaakiaawIcacaGLPaaacqWGKbazcqWG0baDcqGHRaWkdaqadaqaaiabisda0maabmaabaGafmiwaGLbauaadaWgaaWcbaGaeG4mamdabeaakiabgUcaRiabigdaXaGaayjkaiaawMcaamaabmaabaGafmiwaGLbauaadaWgaaWcbaGaeGynaudabeaakiabgUcaRiabicdaWiabc6caUiabiMda5iabikdaYiabiIda4iabiodaZaGaayjkaiaawMcaamaaCaaaleqabaGaeyOeI0IaeGymaedaaOGaey4kaSIaeGOmaiZaaeWaaeaacuWGybawgaqbamaaBaaaleaacqaIXaqmaeqaaOGaey4kaSIaeGimaaJaeiOla4IaeG4naCJaeG4mamJaeG4mamJaeGyoaKdacaGLOaGaayzkaaWaaWbaaSqabeaacqaIYaGmaaaakiaawIcacaGLPaaacqWGKbaziiGacqWFjpWDaeaacqWGKbazcuWGybawgaqbamaaBaaaleaacqaIYaGmaeqaaOGaeyypa0ZaaeWaaeaacqaIXaqmcqaIWaamdaqadaqaaiqbdIfayzaafaWaaSbaaSqaaiabigdaXaqabaGccqGHRaWkcqaIWaamcqGGUaGlcqaI3aWncqaIZaWmcqaIZaWmcqaI5aqoaiaawIcacaGLPaaadaahaaWcbeqaaiabikdaYaaakiabgkHiTiabigdaXiabicdaWmaabmaabaGafmiwaGLbauaadaWgaaWcbaGaeGOmaidabeaakiabgUcaRiabicdaWiabc6caUiabiEda3iabiodaZiabiodaZiabiMda5aGaayjkaiaawMcaamaaCaaaleqabaGaeGOmaidaaaGccaGLOaGaayzkaaGaemizaqMaemiDaqNaey4kaSYaaeWaaeaacqaI2aGndaqadaqaaiqbdIfayzaafaWaaSbaaSqaaiabigdaXaqabaGccqGHRaWkcqaIWaamcqGGUaGlcqaI3aWncqaIZaWmcqaIZaWmcqaI5aqoaiaawIcacaGLPaaadaahaaWcbeqaaiabikdaYaaakiabgUcaRiabiEda3maabmaabaGafmiwaGLbauaadaWgaaWcbaGaeGOmaidabeaakiabgUcaRiabicdaWiabc6caUiabiEda3iabiodaZiabiodaZiabiMda5aGaayjkaiaawMcaamaaCaaaleqabaGaeGOmaidaaaGccaGLOaGaayzkaaGaemizaqMae8xYdChabaGaemizaqMafmiwaGLbauaadaWgaaWcbaGaeG4mamdabeaakiabg2da9maabmaabaGaeGymaeJaeGimaaZaaeWaaeaacuWGybawgaqbamaaBaaaleaacqaIYaGmaeqaaOGaey4kaSIaeGimaaJaeiOla4IaeG4naCJaeG4mamJaeG4mamJaeGyoaKdacaGLOaGaayzkaaWaaWbaaSqabeaacqGHsislcqaIXaqmaaGccqGHsislcqaIXaqmcqaIWaamdaqadaqaaiqbdIfayzaafaWaaSbaaSqaaiabikdaYaqabaGccqGHRaWkcqaIWaamcqGGUaGlcqaI3aWncqaIZaWmcqaIZaWmcqaI5aqoaiaawIcacaGLPaaadaahaaWcbeqaaiabgkHiTiabigdaXaaakmaabmaabaGafmiwaGLbauaadaWgaaWcbaGaeG4mamdabeaakiabgUcaRiabigdaXaGaayjkaiaawMcaamaaCaaaleqabaGaeGOmaidaaaGccaGLOaGaayzkaaGaemizaqMaemiDaqNaey4kaSYaaeWaaeaacqaI1aqndaqadaqaaiqbdIfayzaafaWaaSbaaSqaaiabikdaYaqabaGccqGHRaWkcqaIWaamcqGGUaGlcqaI3aWncqaIZaWmcqaIZaWmcqaI5aqoaiaawIcacaGLPaaadaahaaWcbeqaaiabgkHiTiabigdaXaaakiabgkHiTiabikdaYmaabmaabaGafmiwaGLbauaadaWgaaWcbaGaeGOmaidabeaakiabgUcaRiabicdaWiabc6caUiabiEda3iabiodaZiabiodaZiabiMda5aGaayjkaiaawMcaamaaCaaaleqabaGaeyOeI0IaeGymaedaaOWaaeWaaeaacuWGybawgaqbamaaBaaaleaacqaIZaWmaeqaaOGaey4kaSIaeGymaedacaGLOaGaayzkaaWaaWbaaSqabeaacqaIYaGmaaaakiaawIcacaGLPaaacqWGKbazcqWFjpWDaeaacqWGKbazcuWGybawgaqbamaaBaaaleaacqaI0aanaeqaaOGaeyypa0ZaaeWaaeaacqaI4aaodaqadaqaaiqbdIfayzaafaWaaSbaaSqaaiabiodaZaqabaGccqGHRaWkcqaIXaqmaiaawIcacaGLPaaadaahaaWcbeqaaiabikdaYaaakmaabmaabaGafmiwaGLbauaadaWgaaWcbaGaeGynaudabeaakiabgUcaRiabicdaWiabc6caUiabiMda5iabikdaYiabiIda4iabiodaZaGaayjkaiaawMcaamaaCaaaleqabaGaeyOeI0IaeGymaedaaOGaeyOeI0IaeGymaeJaeGimaaZaaeWaaeaacuWGybawgaqbamaaBaaaleaacqaI0aanaeqaaOGaey4kaSIaeGimaaJaeiOla4IaeGyoaKJaeGOmaiJaeGioaGJaeG4mamdacaGLOaGaayzkaaWaaWbaaSqabeaacqaIYaGmaaaakiaawIcacaGLPaaacqWGKbazcqWG0baDcqGHRaWkdaqadaqaaiabiAda2maabmaabaGafmiwaGLbauaadaWgaaWcbaGaeG4mamdabeaakiabgUcaRiabigdaXaGaayjkaiaawMcaamaaCaaaleqabaGaeGOmaidaaOWaaeWaaeaacuWGybawgaqbamaaBaaaleaacqaI1aqnaeqaaOGaey4kaSIaeGimaaJaeiOla4IaeGyoaKJaeGOmaiJaeGioaGJaeG4mamdacaGLOaGaayzkaaWaaWbaaSqabeaacqGHsislcqaIXaqmaaGccqGHsislcqaIYaGmcqGGUaGlcqaI1aqndaqadaqaaiqbdIfayzaafaWaaSbaaSqaaiabisda0aqabaGccqGHRaWkcqaIWaamcqGGUaGlcqaI5aqocqaIYaGmcqaI4aaocqaIZaWmaiaawIcacaGLPaaadaahaaWcbeqaaiabikdaYaaaaOGaayjkaiaawMcaaiabdsgaKjab=L8a3bqaaiabdsgaKjqbdIfayzaafaWaaSbaaSqaaiabiwda1aqabaGccqGH9aqpdaqadaqaaiabigdaXiabicdaWmaabmaabaGafmiwaGLbauaadaWgaaWcbaGaeGinaqdabeaakiabgUcaRiabicdaWiabc6caUiabiMda5iabikdaYiabiIda4iabiodaZaGaayjkaiaawMcaamaaCaaaleqabaGaeGOmaidaaOGaeyOeI0IaeGymaeJaeGimaaZaaeWaaeaacuWGybawgaqbamaaBaaaleaacqaI1aqnaeqaaOGaey4kaSIaeGimaaJaeiOla4IaeGyoaKJaeGOmaiJaeGioaGJaeG4mamdacaGLOaGaayzkaaWaaWbaaSqabeaacqaIYaGmaaaakiaawIcacaGLPaaacqWGKbazcqWG0baDcqGHRaWkdaqadaqaaiabiIda4maabmaabaGafmiwaGLbauaadaWgaaWcbaGaeGinaqdabeaakiabgUcaRiabicdaWiabc6caUiabiMda5iabikdaYiabiIda4iabiodaZaGaayjkaiaawMcaamaaCaaaleqabaGaeGOmaidaaOGaey4kaSIaeGOnayZaaeWaaeaacuWGybawgaqbamaaBaaaleaacqaI1aqnaeqaaOGaey4kaSIaeGimaaJaeiOla4IaeGyoaKJaeGOmaiJaeGioaGJaeG4mamdacaGLOaGaayzkaaWaaWbaaSqabeaacqaIYaGmaaaakiaawIcacaGLPaaacqWGKbazcqWFjpWDaeaacuWGybawgaqbamaaBaaaleaacqaIXaqmaeqaaOGaeiikaGIaeGimaaJaeiykaKIaeyypa0JaeyOeI0IaeGimaaJaeiOla4IaeGOmaiJaeG4mamJaeG4mamJaeGyoaKJaeiilaWIafmiwaGLbauaadaWgaaWcbaGaeGOmaidabeaakiabcIcaOiabicdaWiabcMcaPiabg2da9iabgkHiTiabicdaWiabc6caUiabikdaYiabiodaZiabiodaZiabiMda5iabcYcaSiqbdIfayzaafaWaaSbaaSqaaiabiodaZaqabaGccqGGOaakcqaIWaamcqGGPaqkcqGH9aqpcqGHsislcqaIWaamcqGGUaGlcqaI1aqncqGGSaalcuWGybawgaqbamaaBaaaleaacqaI0aanaeqaaOGaeiikaGIaeGimaaJaeiykaKIaeyypa0JaeyOeI0IaeGimaaJaeiOla4IaeGinaqJaeGOmaiJaeGioaGJaeG4mamJaeiilaWIafmiwaGLbauaadaWgaaWcbaGaeGynaudabeaakiabcIcaOiabicdaWiabcMcaPiabg2da9iabgkHiTiabicdaWiabc6caUiabisda0iabikdaYiabiIda4iabiodaZiaaxMaacaWLjaGaeiikaGccbaGae4xqaeKae4NmaiJae4xmaeJaeiykaKcaaaa@FA42@

In this stochastic noise case, we can not find a positive definite *P *of the LMIs in (21), so the stability of the stochastic regulatory system is not guaranteed. This result is consistent with the dynamic responses we present in Figures 3(e) and 3(f) for the stochastic system (A20) and the shifted stochastic system in (A21), respectively.

Suppose the genetic regulatory network in (22) suffers an intrinsic stochastic kinetic parameter perturbation as (A18) and an extrinsic noise *υ*(*t*), which is white Gaussian noise with mean = 2 and variance = 1, i.e.,

dX1=(5X3X5−1−10X12+υ1)dt+(X3X5−1−2X12)dωX1(0)=0dX2=(10X12−10X22+υ2)dt+(−2X12+2X22)dωX2(0)=0dX3=(10X2−1−10X2−1X32+υ3)dt+(0.8X2−1−1.4X2−1X32)dωX3(0)=0dX4=(8X32X5−1−10X42+υ4)dt+(1.2X32X5−1−2X42)dωX4(0)=0dX5=(10X42−10X52+υ5)dt+(−1.8X42+X52)dωX5(0)=0     (A22)
 MathType@MTEF@5@5@+=feaafiart1ev1aaatCvAUfKttLearuWrP9MDH5MBPbIqV92AaeXatLxBI9gBaebbnrfifHhDYfgasaacH8akY=wiFfYdH8Gipec8Eeeu0xXdbba9frFj0=OqFfea0dXdd9vqai=hGuQ8kuc9pgc9s8qqaq=dirpe0xb9q8qiLsFr0=vr0=vr0dc8meaabaqaciaacaGaaeqabaqabeGadaaakeaafaqaaeqbcaaaaeaacqWGKbazcqWGybawdaWgaaWcbaGaeGymaedabeaakiabg2da9maabmaabaGaeGynauJaemiwaG1aaSbaaSqaaiabiodaZaqabaGccqWGybawdaqhaaWcbaGaeGynaudabaGaeyOeI0IaeGymaedaaOGaeyOeI0IaeGymaeJaeGimaaJaemiwaG1aa0baaSqaaiabigdaXaqaaiabikdaYaaakiabgUcaRGGaciab=v8a1naaBaaaleaacqaIXaqmaeqaaaGccaGLOaGaayzkaaGaemizaqMaemiDaqNaey4kaSYaaeWaaeaacqWGybawdaWgaaWcbaGaeG4mamdabeaakiabdIfaynaaDaaaleaacqaI1aqnaeaacqGHsislcqaIXaqmaaGccqGHsislcqaIYaGmcqWGybawdaqhaaWcbaGaeGymaedabaGaeGOmaidaaaGccaGLOaGaayzkaaGaemizaqMae8xYdChabaGaemiwaG1aaSbaaSqaaiabigdaXaqabaGccqGGOaakcqaIWaamcqGGPaqkcqGH9aqpcqaIWaamaeaacqWGKbazcqWGybawdaWgaaWcbaGaeGOmaidabeaakiabg2da9maabmaabaGaeGymaeJaeGimaaJaemiwaG1aa0baaSqaaiabigdaXaqaaiabikdaYaaakiabgkHiTiabigdaXiabicdaWiabdIfaynaaDaaaleaacqaIYaGmaeaacqaIYaGmaaGccqGHRaWkcqWFfpqDdaWgaaWcbaGaeGOmaidabeaaaOGaayjkaiaawMcaaiabdsgaKjabdsha0jabgUcaRmaabmaabaGaeyOeI0IaeGOmaiJaemiwaG1aa0baaSqaaiabigdaXaqaaiabikdaYaaakiabgUcaRiabikdaYiabdIfaynaaDaaaleaacqaIYaGmaeaacqaIYaGmaaaakiaawIcacaGLPaaacqWGKbazcqWFjpWDaeaacqWGybawdaWgaaWcbaGaeGOmaidabeaakiabcIcaOiabicdaWiabcMcaPiabg2da9iabicdaWaqaaiabdsgaKjabdIfaynaaBaaaleaacqaIZaWmaeqaaOGaeyypa0ZaaeWaaeaacqaIXaqmcqaIWaamcqWGybawdaqhaaWcbaGaeGOmaidabaGaeyOeI0IaeGymaedaaOGaeyOeI0IaeGymaeJaeGimaaJaemiwaG1aa0baaSqaaiabikdaYaqaaiabgkHiTiabigdaXaaakiabdIfaynaaDaaaleaacqaIZaWmaeaacqaIYaGmaaGccqGHRaWkcqWFfpqDdaWgaaWcbaGaeG4mamdabeaaaOGaayjkaiaawMcaaiabdsgaKjabdsha0jabgUcaRmaabmaabaGaeGimaaJaeiOla4IaeGioaGJaemiwaG1aa0baaSqaaiabikdaYaqaaiabgkHiTiabigdaXaaakiabgkHiTiabigdaXiabc6caUiabisda0iabdIfaynaaDaaaleaacqaIYaGmaeaacqGHsislcqaIXaqmaaGccqWGybawdaqhaaWcbaGaeG4mamdabaGaeGOmaidaaaGccaGLOaGaayzkaaGaemizaqMae8xYdChabaGaemiwaG1aaSbaaSqaaiabiodaZaqabaGccqGGOaakcqaIWaamcqGGPaqkcqGH9aqpcqaIWaamaeaacqWGKbazcqWGybawdaWgaaWcbaGaeGinaqdabeaakiabg2da9maabmaabaGaeGioaGJaemiwaG1aa0baaSqaaiabiodaZaqaaiabikdaYaaakiabdIfaynaaDaaaleaacqaI1aqnaeaacqGHsislcqaIXaqmaaGccqGHsislcqaIXaqmcqaIWaamcqWGybawdaqhaaWcbaGaeGinaqdabaGaeGOmaidaaOGaey4kaSIae8xXdu3aaSbaaSqaaiabisda0aqabaaakiaawIcacaGLPaaacqWGKbazcqWG0baDcqGHRaWkdaqadaqaaiabigdaXiabc6caUiabikdaYiabdIfaynaaDaaaleaacqaIZaWmaeaacqaIYaGmaaGccqWGybawdaqhaaWcbaGaeGynaudabaGaeyOeI0IaeGymaedaaOGaeyOeI0IaeGOmaiJaemiwaG1aa0baaSqaaiabisda0aqaaiabikdaYaaaaOGaayjkaiaawMcaaiabdsgaKjab=L8a3bqaaiabdIfaynaaBaaaleaacqaI0aanaeqaaOGaeiikaGIaeGimaaJaeiykaKIaeyypa0JaeGimaadabaGaemizaqMaemiwaG1aaSbaaSqaaiabiwda1aqabaGccqGH9aqpdaqadaqaaiabigdaXiabicdaWiabdIfaynaaDaaaleaacqaI0aanaeaacqaIYaGmaaGccqGHsislcqaIXaqmcqaIWaamcqWGybawdaqhaaWcbaGaeGynaudabaGaeGOmaidaaOGaey4kaSIae8xXdu3aaSbaaSqaaiabiwda1aqabaaakiaawIcacaGLPaaacqWGKbazcqWG0baDcqGHRaWkdaqadaqaaiabgkHiTiabigdaXiabc6caUiabiIda4iabdIfaynaaDaaaleaacqaI0aanaeaacqaIYaGmaaGccqGHRaWkcqWGybawdaqhaaWcbaGaeGynaudabaGaeGOmaidaaaGccaGLOaGaayzkaaGaemizaqMae8xYdChabaGaemiwaG1aaSbaaSqaaiabiwda1aqabaGccqGGOaakcqaIWaamcqGGPaqkcqGH9aqpcqaIWaamaaGaaCzcaiaaxMaadaqadaqaaGqaaiab+feabjabikdaYiabikdaYaGaayjkaiaawMcaaaaa@382E@

We can calculate the attenuation (amplification) level *ρ*_*o *_of the system by solving the LMIs in (21). After solving the constrained optimization problem in (21), the optimal attenuation level *ρ*_*o *_of genes 1, 2, 3, 4, 5 are 0.5723, 0.5328, 0.5489, 0.6980, 0.6964, for *X*_1 _*X*_2_, *X*_3_, *X*_4 _and *X*_5_, respectively. This means that the noise attenuation levels of these genes can not exceed these values, respectively. The dynamic responses of different *υ*(*t*) are shown in Figure 3(g) and 3(h), from which we can calculate the attenuation levels of system simulation. The results are also consistent with the optimal attenuation levels calculated by the method we proposed.

## Authors' contributions

Bor-Sen Chen gave the topic and developed some results. Yu-Chao Wang performed simulations and evaluated the results.
